# Giant orbital Zeeman effects in a magnetic topological van der Waals interphase

**DOI:** 10.1038/s41467-026-78016-8

**Published:** 2026-10-23

**Authors:** Tobias Wichmann, Mirco Sastges, Keda Jin, Jose Martinez-Castro, Tom G. Saunderson, Dongwook Go, Honey Boban, Samir Lounis, Lukasz Plucinski, Markus Ternes, Yuriy Mokrousov, F. Stefan Tautz, Felix Lüpke

**Affiliations:** 1 Peter Grünberg Institute (PGI-3), Forschungszentrum Jülich, Jülich, Germany; 2 Jülich Aachen Research Alliance, Fundamentals of Future Information Technology, Jülich, Germany; 3 Institut für Experimentalphysik IV A, RWTH Aachen University, Aachen, Germany; 4 Institute of Physics, Johannes Gutenberg University, Mainz, Germany; 5 Peter Grünberg Institute (PGI-1), Forschungszentrum Jülich, Jülich, Germany; 6 Department of Physics, RWTH Aachen University, Aachen, Germany; 7 Institut für Experimentalphysik II B, RWTH Aachen University, Aachen, Germany; 8 Department of Physics, Korea University, Seoul, Republic of Korea; 9 Peter Grünberg Institute (PGI-6), Forschungszentrum Jülich, Jülich, Germany; 10 Institute of Physics and Halle-Berlin-Regensburg Cluster of Excellence CCE, Martin-Luther-University Halle-Wittenberg, Halle, Germany; 11 Institute of Physics II, University of Cologne, Cologne, Germany

**Keywords:** Surfaces, interfaces and thin films, Electronic properties and materials, Magnetic properties and materials

## Abstract

Van der Waals (vdW) heterostructures allow the engineering of magnetic and electronic properties via interfacial orbital hybridization and charge transfer. The resulting electric fields can give rise to Rashba spin-orbit coupling and Dzyaloshinskii-Moriya interactions, which may stabilize novel two-dimensional magnetic phases. While such emergent magnetic ‘interphases’ may offer a promising platform for spin-based electronics, direct spectroscopic evidence for them is still lacking. Here, we report electric-field tunable Zeeman effects with Landé *g*-factors up to  ≈ 230 at the interface of graphene and the vdW ferromagnet Fe_3_GeTe_2_. They arise from an interplay of local-moment canting and the itinerant orbital moments of Fe_3_GeTe_2_, producing a giant asymmetric level splitting. Exploiting the inelastic phonon gap of graphene, scanning tunneling spectroscopy allows us to access the buried interface properties, and quantify Faraday-like screening. Our findings provide new insights and opportunities for electrically controlled interface magnetism, e.g. for spintronics and orbitronics devices.

## Abstract (web summary)

One attractive feature of van der Waals materials is their potential to be combined into sandwich structures that generate unique properties. Here, Wichmann et al proved a clear example of this, demonstrating the emergence of electric field tuneable Zeeman interactions with ultra large *g*-factors at the interface of Fe_3_GeTe_2_ and graphene.

## Introduction

**Fig. 1:**
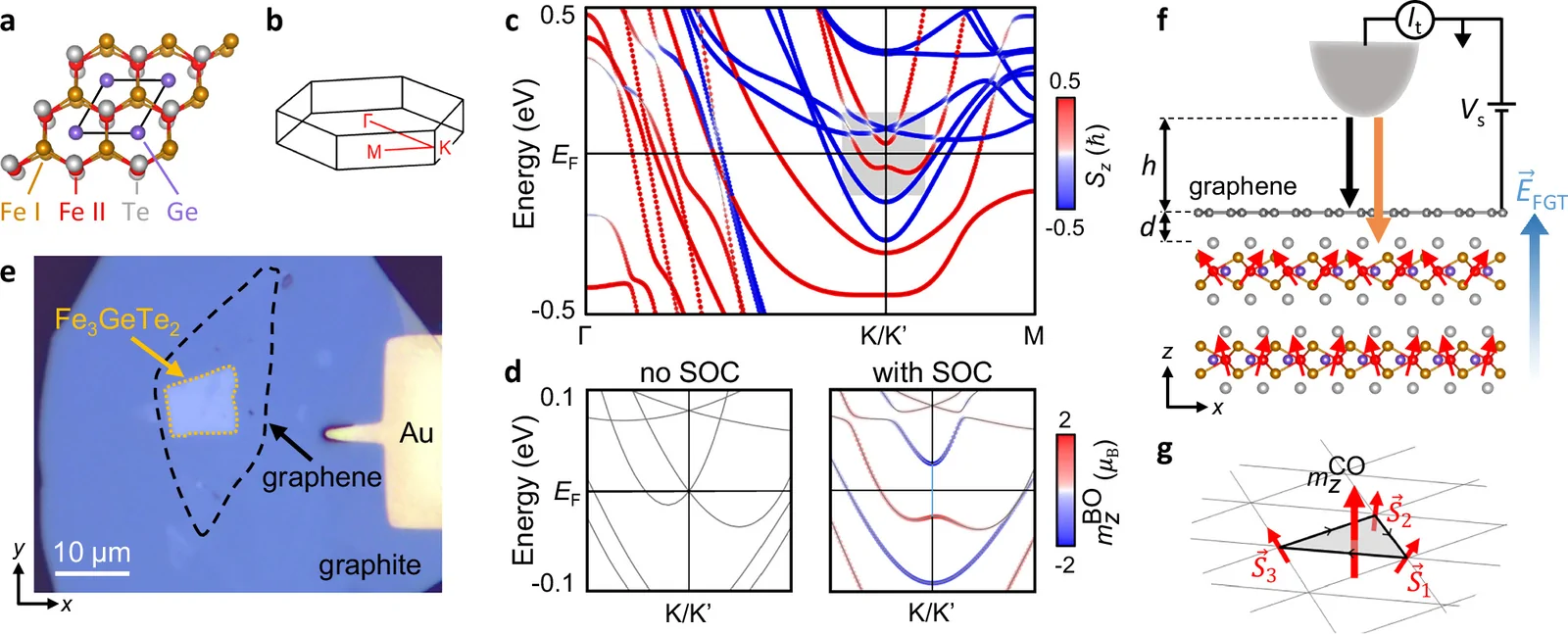
Orbital moments in Fe_3_GeTe_2_ and scanning tunneling microscopy experiment of the graphene/Fe_3_GeTe_2_ heterostructure. **a** Top view of the monolayer Fe_3_GeTe_2_ unit cell, highlighting the two inequivalent Fe sites. **b** First Brillouin zone of Fe_3_GeTe_2_ with indicated symmetry points and lines. **c** Spin-resolved band structure of bulk Fe_3_GeTe_2_, calculated by density functional theory including spin-orbit coupling, with color-coded majority (red) and minority (blue) bands. Near Γ only dispersive bands cross the Fermi energy *E*_F_, while at K/K' several band extrema are located close to *E*_F_. **d** Zoom into the shaded region in panel c, calculated without (left) and with (right) spin-orbit coupling (SOC). The latter calculation shows out-of-plane orbital magnetic moments ${m}_{z}^{{{\rm{BO}}}}$ near the K/K' points. **e** Optical micrograph of a graphene/Fe_3_GeTe_2_ heterostructure. Outlines of the respective flakes are indicated. **f** Schematic side view of the heterostructure and the scanning tunneling microscopy setup. There are two parallel tunneling channels, from the tip to graphene (black arrow) and to Fe_3_GeTe_2_ (orange arrow). The tip-sample distance *h* and graphene-Fe_3_GeTe_2_ distance *d* are indicated. *V*_s_ is the sample bias and *I*_t_ the tunneling current. At the graphene/Fe_3_GeTe_2_ vdW interface, a charge transfer dipole gives rise to an internal electric field ${\vec{E}}_{{{\rm{FGT}}}}$, which results in a canting of the Fe spins at the interface (indicated by the red arrows at the Fe-II sites), due to the Rashba effect and Dzyaloshinskii-Moriya interaction (see main text for details). **g** Schematic of non-collinear spin arrangement in the Fe-I sublattice, where a canting of localized spins ${\vec{S}}_{i}$ results in a conical spin structure. The ensuing scalar spin chirality gives rise to a chiral orbital moment ${m}_{z}^{{{\rm{CO}}}} \sim {\vec{S}}_{1}\cdot ({\vec{S}}_{2}\times {\vec{S}}_{3})$^33–36^. The crystal structures in panels a and f were created using VESTA^75^.

The interplay of topological band structures and magnetism provides a rich playground for the realization of exotic materials^1–3^. This combination of properties may either be achieved by doping magnetism into topological matter, or by employing materials in which intrinsic magnetic order coexists with non-trivial band topologies. An example for the latter case is Fe_3_GeTe_2_ (FGT), a metallic vdW ferromagnet with a Curie temperature of *T*_c_ ≈ 220 K in the bulk^4,5^. While FGT’s magnetic properties at temperatures *T* ≳ *T*_c_ originate from localized Fe moments, for *T* ≪ *T*_c_ itinerant Stoner-like magnetism, carried by coherent delocalized Fe states, dominates^6–8^. In addition to its ferromagnetic properties, the band structure of FGT exhibits a topological nodal line gap that originates from a crossing of hybridized Fe-I and Fe-II spin-majority bands at its **k** = K/K’ points (ref. ^9^ and Fig. 1a–c). The band hybridization near this topological gap results in large Berry curvatures **Ω**(**k**)^9^ and in turn orbital magnetic moments **m**(**k**) ∼ − **Ω**(**k**)^10^ which dominate the spin moments at the edges of these bands (Fig. 1d). The interplay of strongly correlated itinerant magnetism and Fe local moments results in a large magnetic anisotropy energy^11–13^ that allows to control the magnetic state of FGT not only by external magnetic fields but also by charge-addressing stimuli, such as electric fields, currents, and doping^11,14–16^. This opens up the possibility to engineer magnetic properties at interfaces via the electric fields that arise when different materials are brought into contact, or even by external gates.

Here, we report a giant asymmetric Zeeman effect at the interface between monolayer graphene and FGT, which is moreover tunable by varying the interface dipole via electrostatic gating. A detailed analysis combining scanning tunneling microscopy/spectroscopy (STM/STS) and theoretical calculations reveals that this unusual Zeeman effect is the result of two coupled orbital Zeeman effects: On the one hand, the band hybridization near the topological band gap results in localized out-of-plane band orbital moments (BOM) ${m}_{z}^{{{\rm{BO}}}}$ near the K/K’ points. On the other hand, a canting of Fe local moments that has previously been reported in transport studies of graphene/FGT interfaces^13,17^ gives rise to chiral orbital moments (COM) ${m}_{z}^{{{\rm{CO}}}}$, which result from the winding of the itinerant charge carrier wave functions as they traverse the non-collinear texture of localized moments at the vdW interface and which can directly couple to external magnetic fields with large *g*-factors^10,18–20^. This complex compound Zeeman effect signals the emergence of subtle correlations between magnetic and topological electronic properties, which may open up the opportunity to engineer the response of the heterostructure to magnetic and electric fields, e.g., for chiral orbitronics applications.

## Results

### The graphene/FGT heterostructure

**Fig. 2:**
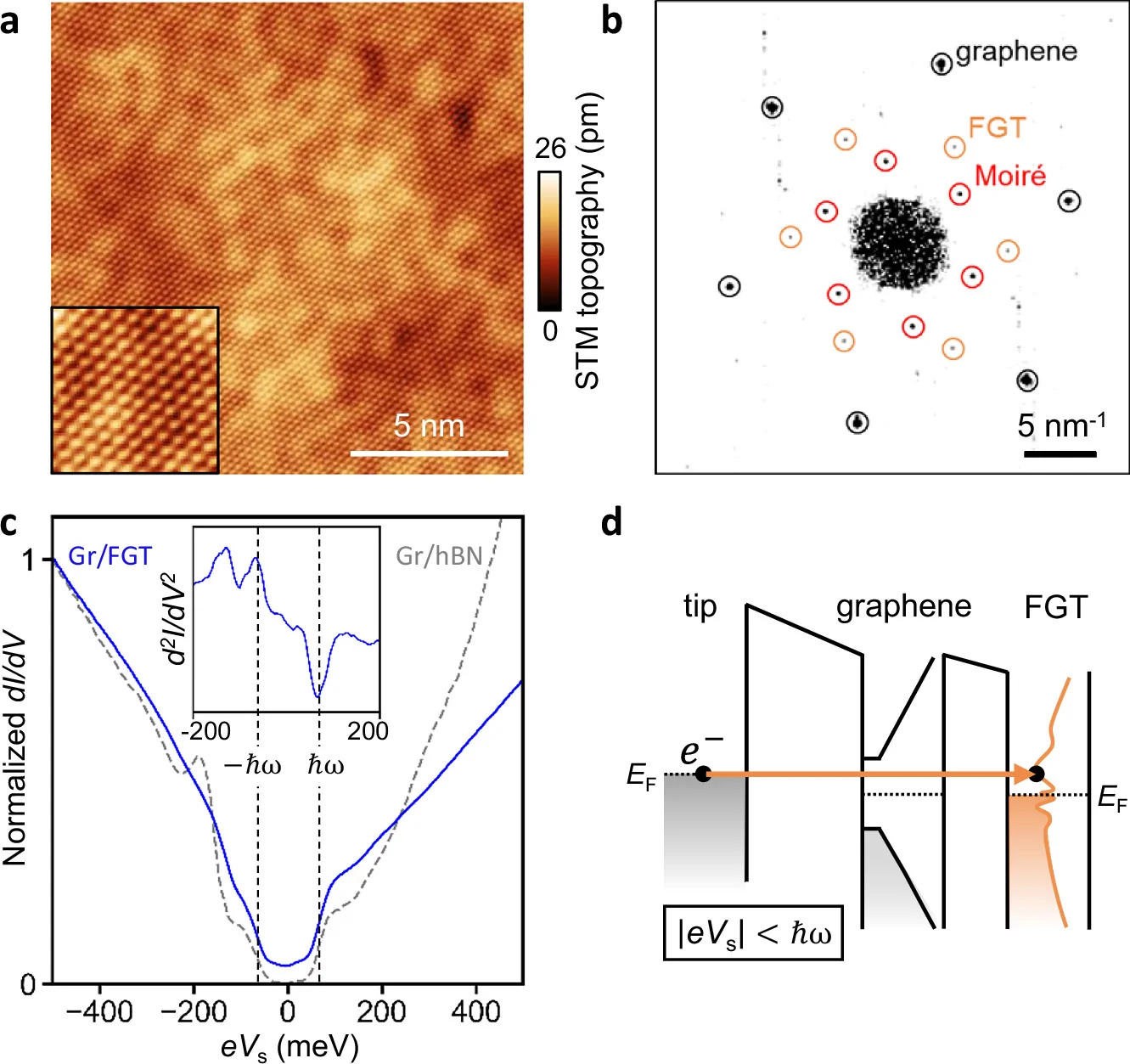
Tunneling through the inelastic gap of graphene. **(**STM topography of the graphene/Fe_3_GeTe_2_ heterostructure surface (*V*_s_ = −50 mV, *I*_t_ = 70 pA), simultaneously revealing atomic resolution of the graphene lattice and a longer-range modulation stemming from the partial occupancy of the Fe-II sites in Fe_3_GeTe_2_. Inset: Zoom into the graphene lattice (scan size 2.5 nm). **b** Fourier transform of the topography in (**a**). The hexagonal patterns of graphene (black circles), Fe_3_GeTe_2_ (orange circles), and their moiré pattern (red circles) are clearly visible. **c** Spatially averaged tunneling conductance spectrum of the heterostructure (blue solid line, *V*_s_ = 500 mV, *I*_t_ = 200 pA). Inset: Second derivative spectrum. Below the inelastic threshold, i.e., for ∣*eV*_s_∣ < *ℏω* where *ω* is the lowest available phonon frequency of graphene, an inelastic tunneling gap is observed (vertical dotted lines), while outside this range the linear dispersion of the graphene Dirac cone is visible. Within the inelastic gap, a significant conductance is measured, in contrast to the findings for graphene/hBN heterostructures (gray dashed line, data from ref. ^24^). The graphene is close to charge neutrality in both cases. For better comparison, both data sets are normalized at *eV*_s_ = −500 meV. **d** Schematic diagram of the tunneling processes. With its gapless spectrum, Fe_3_GeTe_2_ dominates the tunneling current within the inelastic gap of the graphene.

An optical micrograph of a graphene/FGT heterostructure, where a 28 nm-thick FGT flake is encapsulated between monolayer graphene on the top and an additional graphite flake underneath, is shown in Fig. 1e. For details of the sample fabrication see the Methods section. At this thickness and the experimental temperature of ∼6 K, an isolated FGT flake would be in a single-domain ferromagnetic state^14,21^, however, the symmetry breaking and electric field at the interface to the graphene give rise to a Dzyaloshinskii-Moriya interaction, which cants the magnetic moments at the interface away from surface normal^13^, as indicated schematically in the sketch of the heterostructure and the tunneling experiment in Fig. 1f. Constant-current STM topography images of the graphene/FGT heterostructure (Fig. 2a) show a superposition of the atomic lattice of graphene and a long-range modulation that is a characteristic feature of FGT: It stems from random disorder in the partially occupied Fe-II sites^12^. In the corresponding Fourier transform (Fig. 2b), we observe sharp spots due to the graphene (*a*_gr_ = 246 pm) and the FGT lattices (*a*_FGT_ = 399 pm), as well as their Moiré pattern (*a* = 524pm), indicating an atomically clean vdW interface. The relative orientation of both lattices is approximately 17^∘^.

### The inelastic tunneling gap of graphene

Tunneling spectra recorded on the heterostructure reveals the characteristic V-shaped density of states of the graphene Dirac cone (Fig. 2c), as well as an inelastic tunneling gap at the Fermi energy *E*_F_ (*eV*_s_ = 0). This gap results from a suppression of the tunneling probability due to a momentum mismatch between the tunneling tip and the finite parallel momenta of the graphene Dirac states at the K/K’ points (see refs. ^22–24^ and the Methods section for a more detailed discussion). However, above a threshold energy, i.e., for ∣*eV*_s_∣ ≥ *ℏω* = (65 ± 2) meV, phonon-assisted tunneling channels become available, which significantly enhance the tunneling probability. Our observed onset energy of the inelastic tunneling channels corresponds to the lowest-energy phonon mode at the graphene K point^25^, in good agreement with the phonon thresholds reported for graphene on different substrates^22,24,26^. This agreement indicates that the graphene is neither significantly strained nor strongly doped. However, in contrast to graphene on insulating substrates, where the *dI*/*dV* signal almost vanishes below the phonon threshold^22,24,25,27^, we observe a significant tunneling conductance for ∣*eV*_s_∣ < *ℏω*. The reason is that, while tunneling to the graphene is suppressed in this energy range, charge carriers can still tunnel between the tip and the metallic FGT underneath the graphene, which allows us to directly access the electronic properties at the graphene/FGT interface (Fig. 2d and Methods section).

### Giant asymmetric Zeeman effect

**Fig. 3:**
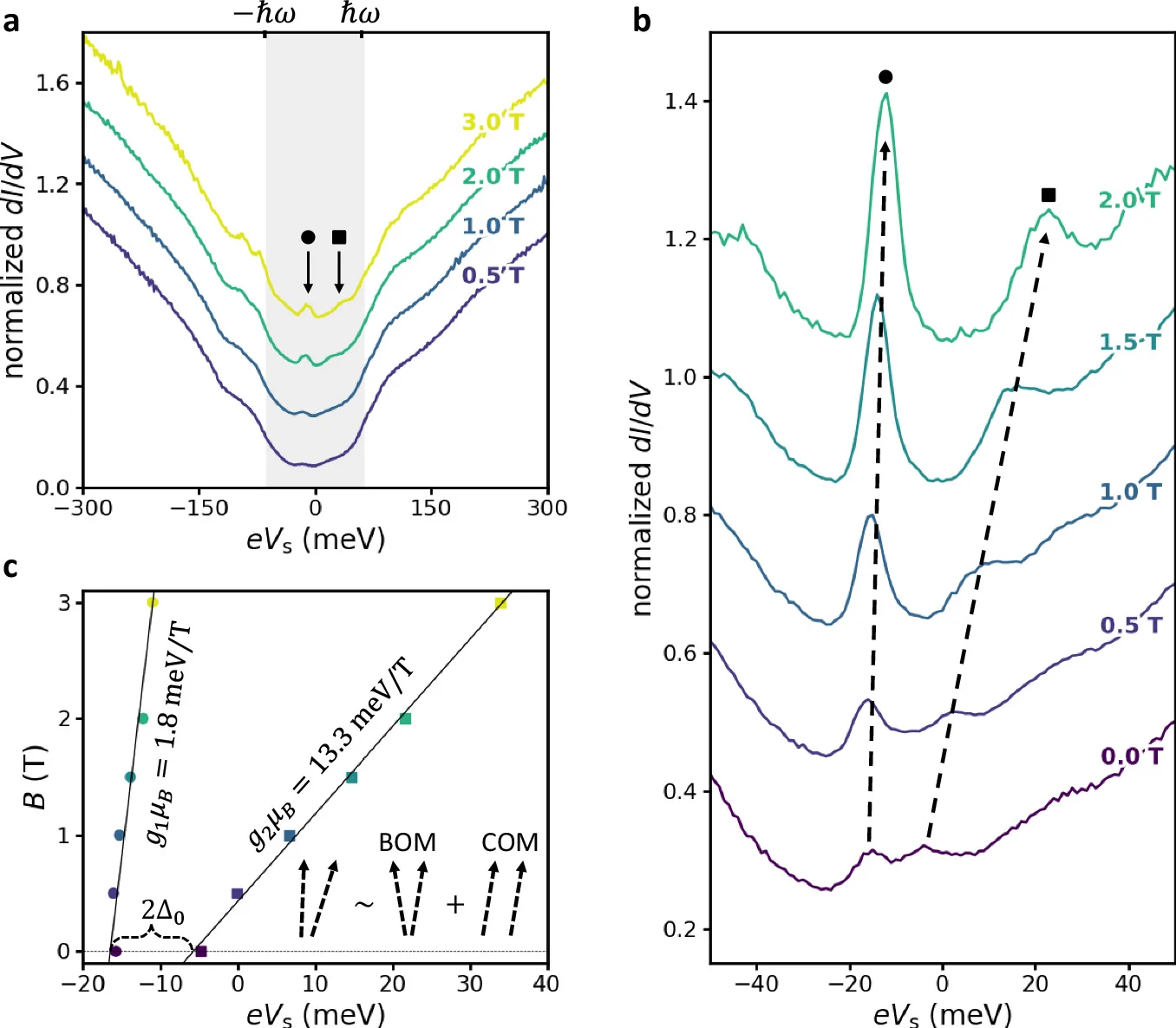
Giant orbital Zeeman effects at the graphene/Fe_3_GeTe_2_ interface. **a** Tunneling spectra (differential conductance in arbitrary units) for different out-of-plane *B* fields (normal to surface). For clarity, curves are offset by 0.2 arb. units each. In the inelastic gap of graphene (shaded region), two peaks emerge with increasing *B* field, as indicated by arrows and symbols. Tip stabilization parameters: *V*_s_ = 300 mV and *I*_t_ = 1 nA. **b** Zoom into the inelastic-gap region, revealing both a continuous increase of the two peaks' intensities and shifts to higher energy as a function of applied *B* field. Curves are offset by 0.2 arb. units each. Dashed arrows are guides to the eye. Tip stabilization parameters: *V*_s_ = 50 mV and *I*_t_ = 0.5 nA. **c** Symbols: Peak positions (horizontal axis) as function of applied *B* field (vertical axis). The solid lines are linear fits, yielding effective Landé *g*-factors *g*_1_ ≈ 30 (circles) and *g*_2_ ≈ 230 (squares). Inset: Schematic decomposition of the observed peak shifts into a splitting and a shifting contribution. The former arises from the band orbital moments (BOM) induced by the topological nodal line gap, while the latter has its origin in the chiral orbital moments (COM) induced by spin canting. For more details, see main text.

A careful analysis of the tunneling spectrum inside the inelastic phonon gap of graphene reveals two peaks, which strengthen on application of a magnetic field (Fig. 3a). At the same time, the linear Dirac spectrum of graphene outside the inelastic tunneling gap remains mostly unaffected. Strikingly, the two peaks within the inelastic gap show significant energy shifts as function of the magnetic field. Linear fits to the data result in *g*_1_*μ*_B_ = (1.8 ± 0.3) meV/T and *g*_2_*μ*_B_ = (13.3 ± 0.4) meV/T, corresponding to Landé *g*-factors *g* = *E*_Zeeman_/(*μ*_B_*B*) of *g*_1_ ≈ 30 and *g*_2_ ≈ 230, respectively (Fig. 3b and Supplementary Fig. S1). Furthermore, a spatial analysis of the peaks shows that they are consistent across the heterostructure surface and not localized at defects (Supplementary Fig. S2), pointing to a band-structure origin of these features. Considering the FGT band structure, the observed peaks can be intuitively understood to stem from the flat band edges near the topological gap at the K/K’ points, where the orbital magnetic moments ${m}_{z}^{{{\rm{BO}}}}$, concentrated at the band extrema, lead to large *g*-factors (Fig. 1 and refs. ^28,29^). This interpretation also explains the increasing peak intensity and sharpness with increasing magnetic field in Fig. 3b, analyzed in detail in Supplementary Fig. S1: DFT calculations show opposite orbital moments at the two band edges (Fig. 1d), such that the topological gap increases as function of the applied magnetic field. Concurrently, the band edges at K/K’ flatten out further, again because the orbital magnetic moment is concentrated at the band extrema (Fig. 1c, d and refs. ^28,29^). Extracting the splitting of the two peaks at *B* = 0 results in 2*Δ*_0_ = (10.8 ± 0.7) meV, which agrees reasonably well with our DFT calculated spin orbit gaps at K/K’ of ∼52 meV and refs. ^9,11^, considering that the gap size decreases when inversion symmetry is broken (Supplementary Fig. S3). Note that the smaller the gap at *B* = 0, the larger the orbital moments ${m}_{z}^{{{\rm{BO}}}} \sim {\Delta }^{-1}$ at K/K’ (see Methods section).

While the model of opposite orbital moments ${m}_{z}^{{{\rm{BO}}}}$, concentrated at the upper and lower band edges, respectively, qualitatively explains both the observation of large *g* factors and of tunneling conductance peaks with increasing intensity and sharpness as the magnetic field is turned up, it disagrees with our experimental observations in that it would predict a *B*-field-induced shift of these conductance peaks in opposite directions, whereas we find that the peaks move in the same direction (to higher energy), albeit with different slopes (Fig. 3c). We therefore conclude that, in addition to the band orbital effect, there must be a second Zeeman effect at play that shifts both conductance peaks to higher energies, as schematically shown in the inset in Fig. 3c. Considering the band structure of FGT, such an effect is indeed expected, because the bands forming the topological gap exhibit the same spin polarization (Fig. 1c and Supplementary Fig. S4). However, including the corresponding spin Zeeman effect, the combined *g*-factor of BOM and the band’s spins at K/K’ is only ${g}_{z}^{{{\rm{BO}}}}=| 2+\langle L\rangle /\langle S\rangle | \lesssim 10$ (ref. ^30^ and Supplementary Fig. S5), where 〈*L*〉 ∼ 3 *ℏ* and 〈*S*〉 ∼ 0.5 *ℏ* are taken from our DFT calculations, corresponding to shifts of  ∼ 0.6 meV/T. Thus, the expected Zeeman effect purely based on FGT’s spin and orbital band magnetism is much too small to explain the experimental observations in Fig. 3.

At this point, we recall that the local Fe moments at the graphene/FGT interface are canted (Fig. 1f), which is not included in the DFT calculations. The reason for the canting is the electric field perpendicular to the interface between the two vdW layers, which gives rise to a Rashba spin-orbit coupling and thus Dzyaloshinskii-Moriya interaction (DMI), which acts on the localized moments of primarily Fe-I $3{d}_{{x}^{2}-{y}^{2}/xy}$ orbitals^8,13,17^. The Rashba coefficient at the graphene/FGT interface was theoretically estimated as *α*_R_ ≈ 246 meVÅ, giving rise to a DMI strength of $| \vec{D}|=0.089$ meV per unit cell^13^, which we expect to be a lower bound for our experiments, because of an enhancement of the interface dipole by the tip compared to experiments discussed in the literature—see the next section. While at intermediate temperatures 10K < *T* < 220 K, this effect leads to skyrmion formation^13^, at our lower experimental temperatures we most likely stabilize a conical phase^31,32^, as schematically shown in Fig. 1f, g.

The un-canting of local moments by the application of an out-of-plane magnetic field is expected to ultimately result in the moments being aligned out-of-plane. However, to fully align the moments, fields much larger than 0.089meV/*μ*_B_ ≳ 1.5 T (ref. ^13^) must be applied, i.e., on the order of 10 T, which are not accessible in our experiment. Also, it cannot explain the observed large *g*-factor of the Zeeman effect in Fig. 3c, because when the moments are canted away from the surface normal and thus are no longer aligned with the *B* field, their Zeeman effect is weakened instead of enhanced. Instead, we propose that the winding of the charge carrier wave functions of the spin-polarized bands that form the topological gap, as they traverse the non-collinear magnetic texture at the vdW interface, gives rise to chiral orbital moments, which are described in detail in the literature^18,19,33–37^. In our system, since the bands in question have identical spin polarization, we expect any local-moment canting-induced COM to affect both bands in the same way, i.e., result in a Zeeman effect that shifts both bands to higher energy as we increase the magnetic field (inset Fig. 3c). Crucially, the canting, and consequently the COM, are the result of the Rashba effect and the resulting DMI at the graphene/FGT interface. Thus, the resulting giant Zeeman effect should be tunable by variation of the electric field across the interface, which we demonstrate in the following.

### Electric field tunability of the Zeeman effect

From simple electrostatic considerations, we estimate the electric field at the graphene/FGT interface as

1 $${E}_{{{\rm{FGT}}}}\equiv {\vec{E}}_{{{\rm{FGT}}}}\cdot \hat{z}=({{{\Phi }}}_{{{\rm{gr}}}}-{{{\Phi }}}_{{{\rm{FGT}}}})/ed\,\approx 1.41\,{{\rm{GV}}}/{{\rm{m}}},$$

with the interlayer distance between graphene and FGT *d* = 3.34 Å (Fig. 1f), the elementary charge *e*, the work function of FGT Φ_FGT_ ≈ 4.08 eV^38^, and ${\Phi}_{\rm{gr}}={\Phi}_{\rm{gr}}^{0} \approx 4.55$ eV^39^ the work function of charge-neutral graphene, respectively. However, when the heterostructure is initially assembled, the work function difference between graphene and FGT gives rise to a charge transfer (electrons flow from the low-work function FGT to the high-work function graphene), resulting in an intrinsically interface-driven *n* doping of the graphene and a corresponding shift of the Dirac point energy ${E}_{{{\rm{D}}}}^{0}$ relative to the Fermi level. Thus the graphene work function becomes ${\Phi}_{\rm{gr}}={\Phi}_{\rm{gr}}^{0}+{E}_{\rm{D}}^{0}$, where ${E}_{{{\rm{D}}}}^{0}$ is a priori unknown.

**Fig. 4:**
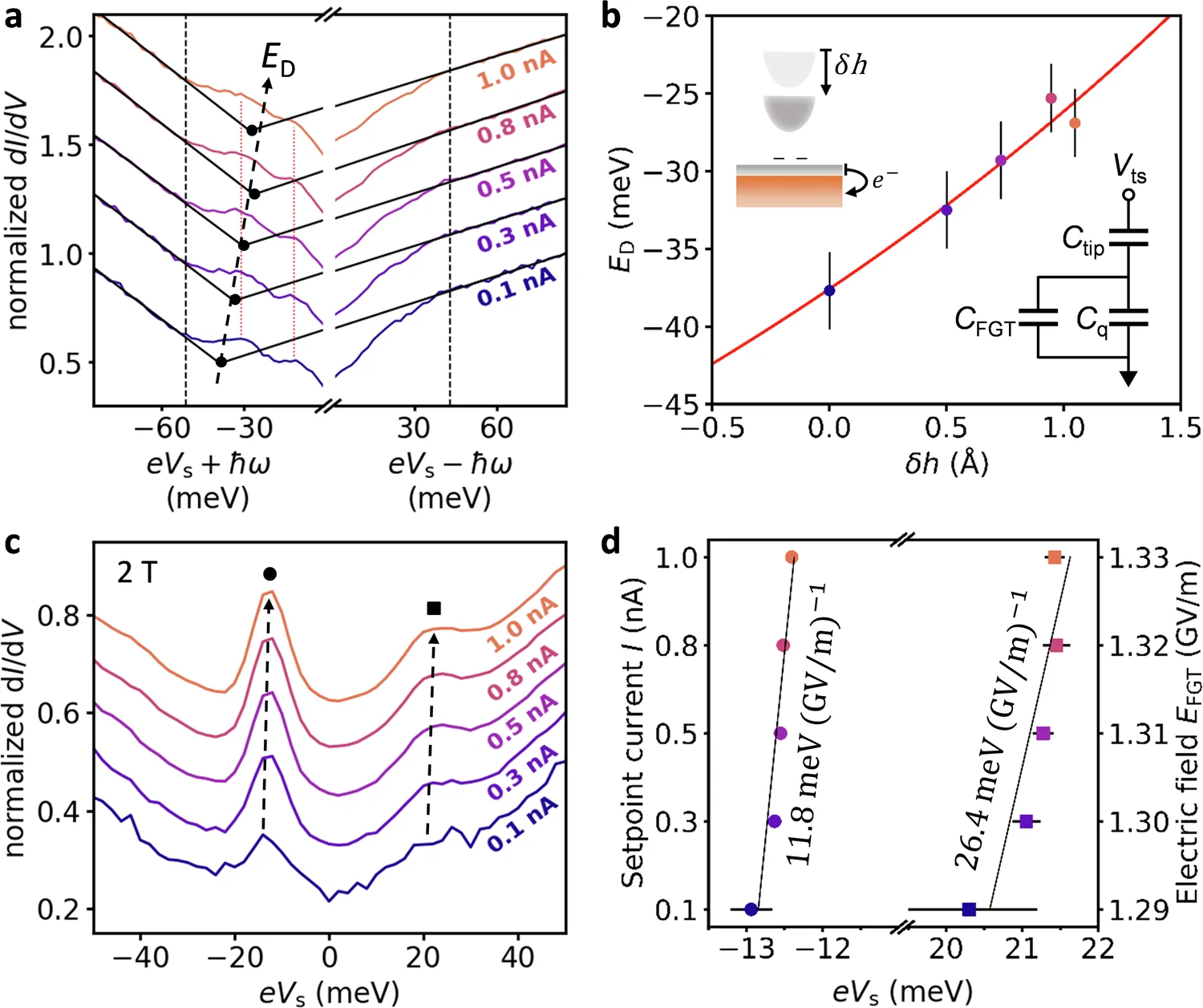
Tuning the orbital Zeeman effects at the graphene/Fe_3_GeTe_2_ interface by electric fields. **a** Differential conductance spectra (in arbitrary units) for different tunneling setpoints. The inelastic gap is cut out, and the curves for different setpoints are offset vertically for clarity. The Dirac point energy *E*_D_ (black dots) is extracted from the crossing points of line fits to the linear graphene spectrum above/below the vertical dashed lines, revealing a shift to higher values as the setpoint is increased. The dashed arrow is a guide to the eye. Remnant features due to Fe_3_GeTe_2_ near the edge of the inelastic gap do not shift with setpoint, as indicated by red dotted lines. The tip stabilization voltage was *V*_s_ = 150 mV for all setpoints. **b** Dirac point energies *E*_D_ (filled circles, extracted from (**a**)) plotted as function of the change in tip-sample distance *δh* (see upper inset). Error bars are propagated from the fits of the Dirac spectrum in panel (a). The red curve is a fit to the data points, using the capacitor model in the lower inset (Eqs. (23) and (24) in the Methods section). **c** Differential conductance spectra (in arbitrary units) of the Fe_3_GeTe_2_-derived peaks in the inelastic gap of graphene for different tunneling setpoints (curves are vertically offset for clarity. All spectra are recorded at a fixed external field of *B* = 2 T. **d** Energy positions of the peaks in panel c as a function of setpoint current. Error bars correspond to the peak fit uncertainties (see Supplementary Fig. S6 for details).

In addition to the interface-driven doping, the presence of the STM tip with its own distinct work function (Φ_tip_ ≈ 4.7 eV, see Methods section) should affect *E*_D_ due to tip gating (clearly, also the applied bias voltage will contribute to a polarity-dependent gating; however, for bias voltages within the inelastic gap of graphene this effect is so small that it can be neglected). In Fig. 4a, tunneling spectra recorded at different setpoints and therefore different tip heights *h* are plotted. Fitting the linear graphene spectrum outside of the inelastic gap to extract the setpoint-dependent Dirac point energy *E*_D_ from the crossing points of the linear fits (Fig. 4a and Supplementary Table 1), we observe a shift of *E*_D_ to more positive values as *h* decreases. This corresponds to the graphene becoming less electron-doped as the tip is brought closer to the sample surface. In contrast, additional FGT features that are located outside the inelastic gap of graphene show no sign of shifting as function of the setpoint current, demonstrating that mainly the graphene doping level is changed by the tip gating (Fig. 4a).

In order to determine the intrinsic doping level of graphene at the interface to FGT, we model the experimental tip-sample geometry by a capacitor circuit (lower inset in Fig. 4b and Methods section), which we fit to the experiment. Extrapolating this model to *δh* → −∞, i.e., to the absence of the tunneling tip, we find a graphene doping level of ${E}_{{{\rm{D}}}}^{0}=-(579\pm 171)$ meV (Fig. 4b). The corresponding intrinsic electric field at the graphene/FGT interface is *E*_FGT_ = (−0.3 ± 0.5) GV/m, i.e., lower and of opposite sign compared to charge-neutral graphene and FGT. We note that a non-zero field is a prerequisite for the anomalous Hall effects and giant magnetoresistances at the graphene/FGT interface reported in the literature^13,40^. In comparison, in the presence of the STM tip the interfacial electric field is significantly larger, in the range *E*_FGT_ = 1.29–1.32 GV/m for tunneling setpoints 0.1−1 nA (see Supplementary Table 1) and thus also the DMI is expected to be larger.

To demonstrate that the variation of the interfacial electric field leads to a variation of the Rashba splitting and DMI, we study the peak positions inside the inelastic gap as function of tunneling setpoint while keeping *B* = 2 T fixed (Fig. 4c). Doing so, we find an increase in the intensity of both peaks as we bring the tip closer to the sample surface. Such behavior is a known effect for tunneling into sharp energy levels originating from states with finite in-plane momenta, such as flat bands and localized states at K/K’^11,41–43^, which again is consistent with our DFT calculations. More interestingly though, extracting the precise peak positions as function of the tunneling setpoint once more reveals systematic energy shifts (Fig. 4d and Supplementary Fig. S6). Linear fits to the data (solid lines in Fig. 4d) are described by *eV*_s_ = (11.8 ± 2.8)meV (GV/m)^−1^*E*_FGT_ − (28.1 ± 2.8) meV and *eV*_s_ = (26.4 ± 8.1) meV (GV/m)^−1^*E*_FGT_ − (13.5 ± 10.6) meV, respectively. Remarkably, the peak positions show a qualitatively similar asymmetric splitting as function of *E* field at fixed *B* field, as they do for the increasing *B* field at fixed *E* field (Supplementary Fig. S7).

### Coupling between band and chiral orbital moments

Our observed DMI-induced Zeeman effect indicates a strong inversion symmetry breaking at the graphene/FGT interface, evidenced by measurements on bulk FGT crystals, where no significant Zeeman effect is observed when the inversion symmetry is only weakly broken (Supplementary Fig. S8a and ref. ^11^). In general agreement with this interpretation, such symmetry breaking has recently been proposed as the origin of the anomalous Hall effect in graphene/FGT ^44^, which can be understood as the manifestation of the Zeeman effect in transport. Our observations imply that the band-structure-induced and chirality-induced moments are directly coupled beyond a mere superposition, which may explain the enhancement of the BOM by more than one order of magnitude compared to the theoretical moments alone. Currently, the exact interaction mechanism between the chiral and BOMs is yet to be understood, but considering that both effects are closely related to the Berry curvature, it does not come as a surprise that they are coupled. Estimating the *g*-factors of the two contributions according to the inset in Fig. 3c results in ${g}_{{{\rm{z}}}}^{{{\rm{BO}}}}\approx 100$ and ${g}_{{{\rm{z}}}}^{{{\rm{CO}}}}\approx 130$, respectively, signifying their similar strength (see Methods section). In this context, we note that the spin Berry curvature vanishes for the band structures shown in Fig. 1c, d. However, spin canting likely leads to a finite spin Berry curvature, with similar localization at the K/K’ points as the Berry curvature^29^, which would couple the spin canting directly to the topological gap. Similar effects were recently reported in magnetic Kagome compounds, where the orbital moments of gapped Dirac cones interact with local moments^18,28,29,45–48^. In the absence of spin canting, these bulk compounds can show spin-orbit-driven orbital Zeeman effects^29,46^, which are initially approximately a linear function of the applied *B* field but saturate as the Berry curvatures decrease with increasing gap sizes. In the presence of spin canting, though, additional orbital moments with *g* ≫ 2 were reported, due an interplay of the spin-orbit gap and spin chirality^10,18,20,28^. However, in contrast to the Kagome compounds, where the Zeeman effects typically saturate at fields of *B* ≲ 2T, we do not observe any sign of a saturation at the graphene/FGT interface up to our maximum accessible field of 3 T, which is consistent with the spin un-canting at the graphene/FGT occuring only at much larger fields *B* ∼ 10 T.

In conclusion, despite the lack of strong bonding in graphene/FGT vdW heterostructures, interface dipoles give rise to emergent magnetoelectronic properties, which are localized at the 2D interface and result in a rich interplay of orbital magnetism and local moments. The required interfacial fields are readily achievable in state-of-the-art electronic devices^49–51^, highlighting the technological potential of our findings. Thus, the present vdW interphase provides a flexible platform to engineer magnetic properties, allowing to control them via gate-tuning the Rashba effect and DMI, which may find applications in spintronics or orbitronics.

## Methods

### Density functional theory

The orbital magnetization in three-dimensional *k*-space is given by ref. ^10^

2 $${{\bf{M}}}^{{\rm{BO}}}=	\dfrac{e}{2\hslash}{{\rm{Im}}}{\sum }_{n}\int_{{\rm{BZ}}}\dfrac{d{{\bf{k}}}}{\left(2\pi \right)^{3}}\,{f}_{n{{\bf{k}}}}\left\langle {\partial}_{{\bf{k}}}{u}_{n{{\bf{k}}}}\right\vert \times \left({\hat{H}}_{{\bf{k}}}+{\varepsilon}_{n{{\bf{k}}}}-2\mu \right)\\ 	 \left\vert {\partial}_{{\bf{k}}}{u}_{n{{\bf{k}}}}\right\rangle,$$

where $\left\vert {u}_{n{{\bf{k}}}}\right\rangle$ is the Bloch-like eigenstate associated with the *n*^th^ eigenvalue *ε*_*n***k**_ of the Hamiltonian *H*_**k**_ of the system, *f*_*n***k**_ is the zero-temperature Fermi occupation factor and *μ* the chemical potential. Note that the vector product is taken between the two derivatives ${\partial }_{{{\bf{k}}}}=\frac{\partial }{\partial {{\bf{k}}}}$ in the bra and ket, respectively.

Unlike transport, which probes all occupied states and is therefore sensitive to the orbital magnetization, STM/S probes the either filled or empty states at a given energy and is thus sensitive to the orbital magnetic moment (OMM) of the bands, which is given by

3 $${m}_{n}{({{\bf{k}}})}^{{{\rm{BO}}}}=\dfrac{e}{2\hslash }{{\rm{Im}}}\langle {\partial }_{{{\bf{k}}}}{u}_{n{{\bf{k}}}}| \times \left({\hat{H}}_{{{\bf{k}}}}-{\varepsilon }_{n{{\bf{k}}}}\right)| {\partial }_{{{\bf{k}}}}{u}_{n{{\bf{k}}}}\rangle .$$

To calculate this quantity using ab initio methods, we performed the following procedure: The electronic structure of bulk Fe_3_GeTe_2_ was computed with the density functional theory code FLEUR^52^ using the full-potential linearized augmented plane wave method^53^. We chose the Perdew-Burke-Ernzerhof exchange correlation functional within the generalized gradient approximation^54^. The FGT lattice parameters are *a* = 3.99 Å and *c* = 16.33 Å in a hexagonal close-packed lattice^55^. We performed a full self-consistent calculation to find the charge density including the effect of spin-orbit coupling within FLEUR, with a resulting magnetocrystalline anisotropy of 1.06 meV per Fe atom. The plane-wave cutoffs for the basis functions, for the charge density and for the exchange correlation functional were set to $5{a}_{0}^{-1}$, $15{a}_{0}^{-1}$, and $12.5{a}_{0}^{-1}$, respectively, where *a*_0_ is the Bohr radius. For Fe and Ge, the maximum in the angular momentum expansion was set to ${l}_{\max }=8$ and the muffin-tin radii *r*_MT_ for both species was set to 2.12*a*_0_; for Te, ${l}_{\max }=10$ and *r*_MT_ = 2.74 *a*_0_. Despite previous work on FGT indicating the effects of strong electronic correlations^7,12^, in the present scenario we do not see indications of such correlations, e.g., as the conduction band minimum is moved through the Fermi energy, and thus did not consider them theoretically. Using the Wannier90 package^56,57^, from the DFT-calculated Bloch wave functions $\left\vert {u}_{n{{\bf{k}}}}\right\rangle$, we constructed maximally localized Wannier functions (MLWFs) and determine the Bloch-like basis $\left\vert {u}_{n{{\bf{k}}}}^{{{\rm{W}}}}\right\rangle$^58,59^. A mesh of 8 × 8 × 8 *k*-points was used with 192 Bloch states to obtain 96 MLWFs. Initial projections were chosen to be *d* states for Fe and *p* states for Ge and Te atoms. The maximum frozen window was set to 2.344 eV above the Fermi energy. The Hamiltonian, spin, and orbital angular momentum (OAM) operators were evaluated in the Bloch basis and transformed into the MLWF basis. The OAM was integrated inside the muffin-tins. Further details of the calculations can be found in Ref. ^60^.

In the Wannier description, the orbital magnetization can be quantified as proposed by Lopez et al.^59^: First, the Bloch-like basis $\left\vert {u}_{n{{\bf{k}}}}^{{{\rm{W}}}}\right\rangle$ is transformed into the so called Hamiltonian gauge, using the transformation

4 $$\left\vert {u}_{n{{\bf{k}}}}^{{{\rm{H}}}}\right\rangle=\mathop{\sum}\limits_{m}^{J}\left\vert {u}_{m{{\bf{k}}}}^{{{\rm{W}}}}\right\rangle {U}_{mn}\left({{\bf{k}}}\right).$$

This is necessary in order to compute the magnetization according to the so-called “modern theory of orbital magnetization” expression Eq. (2). This expression allowed us to decompose the orbital magnetization into its local

5 $${{{\bf{M}}}}_{{{\rm{LC}}}}^{{{\rm{BO}}}}=\dfrac{e}{2\hslash }{{\rm{Im}}}\mathop{\sum}\limits_{n}\int_{{{\rm{BZ}}}}\dfrac{d{{\bf{k}}}}{{\left(2\pi \right)}^{3}}\,{f}_{n{{\bf{k}}}}\langle {\partial }_{{{\bf{k}}}}{u}_{n{{\bf{k}}}}^{{{\rm{H}}}}| \times \left({\hat{H}}_{{{\bf{k}}}}-\mu \right)| {\partial }_{{{\bf{k}}}}{u}_{n{{\bf{k}}}}^{{{\rm{H}}}}\rangle,$$

and itinerant contributions

6 $${{{\bf{M}}}}_{{{\rm{IC}}}}^{{{\rm{BO}}}}=\dfrac{e}{2\hslash }{{\rm{Im}}}\mathop{\sum}\limits_{n}\int_{{{\rm{BZ}}}}\dfrac{d{{\bf{k}}}}{{\left(2\pi \right)}^{3}}\,{f}_{n{{\bf{k}}}}\langle {\partial }_{{{\bf{k}}}}{u}_{n{{\bf{k}}}}^{{{\rm{H}}}}| \times \left({\varepsilon }_{n{{\bf{k}}}}-\mu \right)| {\partial }_{{{\bf{k}}}}{u}_{n{{\bf{k}}}}^{{{\rm{H}}}}\rangle .$$

We computed the band-resolved contributions to the magnetization

7 $${m}_{n,{{\rm{LC}}}}^{{{\rm{BO}}}}=\dfrac{e}{2\hslash }{{\rm{Im}}}\langle {\partial }_{{{\bf{k}}}}{u}_{n{{\bf{k}}}}^{{{\rm{H}}}}| \times \left({\hat{H}}_{{{\bf{k}}}}-\mu \right)| {\partial }_{{{\bf{k}}}}{u}_{n{{\bf{k}}}}^{{{\rm{H}}}}\rangle,$$

and

8 $${m}_{n,{{\rm{IC}}}}^{{{\rm{BO}}}}=\dfrac{e}{2\hslash }{{\rm{Im}}}\langle {\partial }_{{{\bf{k}}}}{u}_{n{{\bf{k}}}}^{{{\rm{H}}}}| \times \left({\varepsilon }_{n{{\bf{k}}}}-\mu \right)| {\partial }_{{{\bf{k}}}}{u}_{n{{\bf{k}}}}^{{{\rm{H}}}}\rangle,$$

similar to ref. ^59^, where it was shown that Eqs. (5) and (6) can be recast into a gauge-invariant formulation. A more detailed theoretical description will be presented in a forthcoming publication. Finally the orbital magnetic moments in Eq. (3) were determined from the *k*-resolved local and itinerant contributions as ${m}_{n}{({{\bf{k}}})}^{\rm{BO}}={m}_{n,{\rm{LC}}}^{\rm{BO}}({\bf{k}}) - {m}_{n,{\rm{IC}}}^{\rm{BO}}({\bf{k}})$.

We note at this point that an earlier proposed model of the relevant bands at K/K’^9^, when inserted into Eq. (3), results in orbital moments which directly contradict our DFT calculations, indicating that such a model does not seem suitable to capture the full orbital physics.

### Sample fabrication

Graphite and FGT crystals were exfoliated onto SiO_2_ and assembled by a standard dry-transfer technique with a microdome polyvinylchlorid/polydimethylsiloxane (PVC/PDMS) stamp^61^. In the resulting heterostructure, the encapsulation of FGT by the larger graphene and graphite flake prevents the former’s degradation. Ti/Au contacts were fabricated using maskless optical lithography, and samples were subsequently cleaned in an ambient-condition atomic force microscope by repeated scanning in contact mode overnight. Supplementary Fig. S9 shows an AFM image, recorded before the STM measurements, of the heterostructure displayed in Fig. 1e. After introduction into the STM ultra-high vacuum chamber, the sample was annealed at 240 ^∘^C for 15 min to desorb residues and water and subsequently transferred to the STM. There, the sample was zero-field cooled to the base temperature of ∼6.1 K, and magnetized by ramping the surface-normal field to *B* = 2 T, prior to STS measurements.

Graphite and Fe_3_GeTe_2_ were obtained from commercial sources (graphite from HQ graphene, FGT from 2D semiconductors). FGT bulk crystals from the same batch were characterized by angle-resolved photoemission spectroscopy (ARPES), showing general agreement with the DFT calculations and literature, and a doping level for which *E*_F_ is close to the topological gap (Supplementary Fig. S8b). STM/S spectra taken on the bulk crystals did not exhibit any peak features and virtually no magnetic field dependence (Supplementary Fig. S8a), confirming the graphene/FGT interface as the source of the observed behavior.

### Scanning tunneling experiments

All STM/STS measurements were performed in a commercial Sigma Polar instrument at ∼6.1 K. Electro-chemically etched W tips were annealed to orange glow in vacuum and prepared on an Ag(111) surface. After introducing the heterostructure sample to the STM scanner, we first approached the tip to the contact leads using visual control and then navigated the tip to the heterostructure by scanning. On the heterostructure, we performed large-area STM scans to locate the graphene and FGT flakes and to check the cleanliness of the heterostructure surface. To ensure a clean tunneling spectrum, we then moved the tip back to the Ti/Au contact and safeguarded that it showed a linear *I*(*V*) characteristics by repeated careful indentation. We then retracted the tip out of tunneling contact and jumped directly back to the clean surface of the heterostructure, where we performed the measurements, thus preventing any tip alterations that may otherwise occur during intermediate scans. All spectroscopy measurements were performed after ramping the magnetic field above the saturation field of the FGT flake, to achieve a well-defined magnetization. STS was performed using standard lock-in techniques at a frequency of 877 Hz and modulation amplitudes of ${V}_{{{\rm{s}}}}^{{{\rm{mod}}}}=1\,{{\rm{to}}}\,\,3$ meV. STM topographs were analyzed using Gwyddion^62^.

The trapezoidal shape of the biased tunneling barrier in the WKB-approximation results in a non-linear dependence of the tunneling conductance as the voltage is increased:

9 $$dI/dV\propto \rho \,{e}^{-2\kappa (E)z},$$

with the density of states *ρ*, the decay constant

10 $$\kappa (E)=\sqrt{\dfrac{2m}{{\hslash }^{2}}({\Phi}_{\rm{eff}} - E)+{k}_{\parallel }^{2}},$$

the effective barrier height

11 $${{{\Phi }}}_{{{\rm{eff}}}}=\dfrac{{{{\Phi }}}_{{{\rm{tip}}}}+{{{\Phi }}}_{{{\rm{sample}}}}}{2}$$

for tunneling bias *E* = *eV*_s_ ≪ Φ_eff_, and the momentum in the surface plane *k*_∥_. As a result, states close to *E*_F_ tend to have a smaller *dI*/*dV* intensity than those at higher bias. In graphene, the intensity difference between states at lower and higher tunneling bias is further enhanced by the inelastic tunneling channels that become available at ∣*eV*∣ > *ℏω = E*_ph_, which significantly increase the tunneling probability^22^. Inside this inelastic gap, the elastic tunneling current to the graphene is small, such that tunneling from the FGT dominates: Although the FGT is further away from the tip than the graphene and thus has lower tunneling probability (Eq. (9)), because of its significantly higher density of states compared to the graphene, it dominates the tunneling for ∣*eV*∣ < *E*_ph_: We estimate the respective elastic tunneling probabilities by evaluating Eqs. (9-11) at the Fermi energy (*E* = 0). For graphene (*k*_∥_ = 1.7 Å^−1^ and *z* = *h* = 10 Å), it results *e*^−2*κh*^ ≈ 3 × 10^−18^. For FGT on the other hand (*k*_∥_ = 1.05 Å^−1^ and *z* = *h* + *d* = 13.34 Å), it results *e*^−2*κ*(*h*+*d*)^ ≈ 5 × 10^−18^. Thus, the theoretical elastic tunneling probability to the K/K'-point states in the FGT is larger than in the graphene. In addition, since FGT has a significantly higher density of states than graphene, because the Fe 3*d* bands near K/K' are flatter than the graphene *π*-bands, we conclude that the tunneling inside the inelastic gap is dominated by the FGT.

We further note that, because the graphene is in direct contact to the extended metallic FGT flake, dynamical Coulomb blockade effects are strongly suppressed in our experiments^63^ and thus cannot explain our observed gap features. This argument is supported by the fact that the observed inelastic gaps are almost identical compared to graphene on insulating substrates^22,24,26^.

The combined observation of a finite peak splitting at zero field followed by an approximately linear shift as function of magnetic field effectively rules out Landau level formation in the graphene as a potential origin of the peak features, since they would follow a square-root dependence $E\propto \sqrt{B}$ in the absence of lateral confinement^64^. We expect lateral confinement to be weak in our heterostructure, because the graphene is coupled to the metallic FGT, which renders it *n*-doped everywhere. Furthermore, our observations are not consistent with conventional quantum-dot (QD) bound states or whispering gallery modes (WGMs): Such states typically give rise to a series of peaks in the density of states corresponding to bound states with increasing (angular) momentum quantum numbers^65,66^. In contrast, our heterostructure exhibits only two well-isolated peak features within the inelastic gap. These features instead are phenomenologically much more similar to tunable orbital moments of a gapped graphene Dirac cone^67,68^. In those works, lateral carrier confinement in electrostatically defined potential wells lifts the graphene’s inversion symmetry and gives rise to orbital magnetic moments, which couple to external electric and magnetic fields. In contrast to this work however, in our heterostructure, both, inversion and time-reversal symmetry are already broken, such that we do not require lateral confinement to realize field tunable orbital moments. Instead, we observe the orbital moments of the gapped FGT nodal line, which dominates the tunneling. This interpretation is supported by the fact that the states intrinsic to the graphene shift according to the graphene doping level^68^, whereas the peaks in our experiments do not: In Fig. 4a, we demonstrate that the graphene doping level can be tuned by Δ*E*_D_ ∼ 15 meV, by changing the tip-sample distance. However, the peaks inside the inelastic gap only shift asymmetrically by ≲ 1 meV, which points to their origin not in the graphene, but in the FGT. This interpretation is again consistent with our argument above, that tunneling inside the inelastic gap is dominated by the FGT. Lastly, we rule out the formation of a quantum dot in the FGT itself, because of its metallic nature.

We extracted *E*_D_ in Fig. 4a from the V-shaped Dirac spectrum outside the inelastic gap by (i) subtracting the energy offset resulting from the inelastic tunneling process above the phonon threshold ±*ℏω*, for positive and negative bias voltages separately, and (ii) performing linear fits to the electron and hole sides of the linear Dirac cone spectrum, respectively; the Dirac point energy *E*_D_ at each setpoint current was then extracted from their intersection. To determine *δh* in Fig. 4, we used the exponential dependence of the tunneling current on the tip-sample distance $I/{I}_{0}=\exp (\frac{{h}_{0}-h}{\lambda })$, where *δh* = *h*_0_ − *h* and *λ*^−1^ ≈ (0.455 Å)^−1^ is the decay constant of the tunneling current for monolayer graphene above the phonon threshold^22^. Then

12 $$\delta h=\lambda \ln (I/{I}_{0}),$$

which is independent of the absolute values of *h* and *h*_0_.

### Electrostatic model

From the experimental geometry, we estimate the tip and FGT capacitance density with respect to the graphene as

13 $${C}_{{{\rm{tip}}}}=\dfrac{{\varepsilon }_{0}}{h}\approx 0.88\,\upmu {\rm{Fc{m}}}^{-2}$$

14 $${C}_{{{\rm{FGT}}}}=\dfrac{{\varepsilon }_{0}}{d}\approx 2.651\,\upmu {\rm{Fc{m}}}^{-2},$$

where *ε*_0_ is the vacuum permittivity and we estimate a graphene/FGT layer spacing of *d* = 3.34 Å and the initial tip height of *h* = 10 Å at setpoint *V*_s_ = 300 mV, *I*_0_ = 0.1 nA. We note that, realistically, the permittivity of the graphene/FGT interface is expected to be higher than *ε*_0_, which would result in an increase of *C*_FGT_ and thus a decrease of the electric field at the graphene/FGT interface. The finite screening length of the electric field in the FGT, on the other hand, leads to a decrease of *C*_FGT_; however, this change is expected to be small because of the metallic character of FGT and the resulting short screening length^69^. In any case, both of these effects have only minor influence on the tip-height dependence of the interface field in our study, because the response of the system is dominated by the change of the tip-sample capacitance *C*_tip_.

To model the charge distribution across the junction and the heterostructure, we must take into account that the graphene screens the tip electric field from the FGT, similar to a Faraday screen^70^. We therefore employed a capacitor model of the junction similar to the one used in ref. ^71^. Because of the low density of states of graphene close to the Dirac point, the model needs to include the graphene quantum capacitance (per unit sample area)^72^

15 $${C}_{{{\rm{q}}}}={e}^{2}\rho,$$

where

16 $$\rho=\dfrac{2{E}_{{{\rm{D}}}}}{\pi {(\hslash {v}_{{{\rm{F}}}})}^{2}}$$

is the spin- and valley-degenerate graphene density of states at *E*_F_ (per unit sample area), and *v*_F_ the Dirac Fermi velocity. Integrating *ρ* from the Dirac point to *E*_F_, one obtains from Eq. (16) the number of carriers per unit area in graphene as ${n}_{{{\rm{gr}}}}={\pi }^{-1}{({E}_{{{\rm{D}}}}/\hslash {v}_{{{\rm{F}}}})}^{2}$. As usual, the quantum capacitance is implemented as a capacitor in series to the geometric capacitance between tip and graphene, *C*_tip_ (see inset Fig. 4b)^71^. As a result of the quantum capacitance, the tip electric field is not perfectly screened by the graphene and can penetrate to the FGT underneath, establishing a capacitance *C*_FGT_ between graphene and FGT, in series with *C*_tip_. Because it is equivalent to a single capacitor with distance *h* + *d*, the arrangement of *C*_tip_ and *C*_FGT_ in series is consistent with a tunneling path from the tip directly to the FGT. We thus have two parallel tunneling paths (the other one from the tip to graphene), which suggests putting the two capacitances *C*_q_ and *C*_FGT_ in parallel, both connected to the same back contact of the Ti/Au substrate (within the metallic FGT layer, there will be no potential drop, despite its considerable thickness). In this model, we neglect the inhomogeneity of the electric field due to the curved tip shape, because we are interested only in the properties right underneath the tip apex and the small graphene-FGT distance compared to the tip radius (10–100 nm) results in effectively parallel electric field lines there.

From the circuit diagram it is clear that

17 $$\dfrac{{n}_{{{\rm{FGT}}}}e}{{C}_{{{\rm{FGT}}}}}=\dfrac{{n}_{{{\rm{gr}}}}e}{{C}_{{{\rm{q}}}}}=\dfrac{{n}_{{{\rm{tip}}}}e}{{C}_{{{\rm{tip}}}}}-{V}_{{{\rm{ts}}}}$$

and

18 $${n}_{{{\rm{tip}}}}+{n}_{{{\rm{gr}}}}+{n}_{{{\rm{FGT}}}}=0,$$

the latter due to charge conservation, where *n*_gr_, *n*_tip_, and *n*_FGT_ are the areal charge densities on graphene and the surfaces of the tip and FGT, respectively. We solve the linear equation system by substituting expressions derived from Eq. (17) into Eq. (18), obtaining

19 $${n}_{{{\rm{gr}}}}+\dfrac{{C}_{{{\rm{tip}}}}{V}_{{{\rm{ts}}}}}{e}+\dfrac{{C}_{{{\rm{tip}}}}}{{C}_{{{\rm{q}}}}}{n}_{{{\rm{gr}}}}+\dfrac{{C}_{{{\rm{FGT}}}}}{{C}_{{{\rm{q}}}}}{n}_{{{\rm{gr}}}}=0.$$

Using the graphene quantum capacitance (Eqs. (15) and (16))^72^

20 $${C}_{{{\rm{q}}}}=\dfrac{2{e}^{2}}{\sqrt{\pi {(\hslash {v}_{{{\rm{F}}}})}^{2}}}\sqrt{{n}_{{{\rm{gr}}}}},$$

results in

21 $${n}_{{{\rm{gr}}}}+({C}_{{{\rm{tip}}}}+{C}_{{{\rm{FGT}}}})\dfrac{\sqrt{\pi {(\hslash {v}_{{{\rm{F}}}})}^{2}}}{2{e}^{2}}\sqrt{{n}_{{{\rm{gr}}}}}+\dfrac{{C}_{{{\rm{tip}}}}{V}_{{{\rm{ts}}}}}{e}=0.$$

Substituting $$a\equiv \frac{\sqrt{\pi {(\hslash {v}_{{{\rm{F}}}})}^{2}}}{2{e}^{2}}$$ gives

22 $${n}_{{{\rm{gr}}}}+a({C}_{{{\rm{tip}}}}+{C}_{{{\rm{FGT}}}})\sqrt{{n}_{{{\rm{gr}}}}}+\dfrac{{C}_{{{\rm{tip}}}}{V}_{{{\rm{ts}}}}}{e}=0,$$

which yields

23 $$\sqrt{{n}_{{{\rm{gr}}}}}=	 -\dfrac{a({C}_{{{\rm{tip}}}}+{C}_{{{\rm{FGT}}}})}{2}\\ 	+\sqrt{{\left(\dfrac{a({C}_{{{\rm{tip}}}}+{C}_{{{\rm{FGT}}}})}{2}\right)}^{2}-\dfrac{{C}_{{{\rm{tip}}}}{V}_{{{\rm{ts}}}}}{e}},$$

where only the positive solution of the quadratic expression is physical, as *n*_gr_ must vanish at *V*_ts_ = 0. With Eq. (23), we determined the Dirac point energy as

24 $${E}_{{{\rm{D}}}}=\pm {2e}^{2}a\sqrt{{n}_{{{\rm{gr}}}}}.$$

The positive (negative) sign applies to holes (electrons).

The potential difference *V*_ts_ between the tip and graphene is given by the sum of the applied bias voltage and the contact potential difference between tip and sample surface

25 $${V}_{\rm{ts}}={V}_{\rm{s}}+{V}_{\rm{CPD}}={V}_{\rm{s}}+({\Phi}_{\rm{tip}} - {\Phi}_{\rm{gr}})/e$$

where the Φ are the respective work functions. While the work function of charge-neutral graphene is well known (see above), the work function of the tip is more difficult to estimate, and critically depends on the details of the tip apex. As a result, for W tips the tip work function typically varies in the range 4.5−5.1 eV^11^. We indent our W tips into Au before measurements and expect Au to stick to the tip as a result of the preparation procedure. The work function of bulk Au is Φ_Au_ ≈ 5.1 eV, such that we estimate the resulting tip work function to be in the range Φ_tip_ = 4.6 −5.1 eV, i.e., greater or equal to that of graphene. The fit in Fig. 4b results in a tip work function of Φ_tip_ = (4.66 ± 0.03) eV, in excellent agreement with this range of expected values. Our model further compares well with experiments on graphene/NbSe_2_ heterostructures (${\Phi}_{{\rm{NbSe}}_{2}}$ ≈ 5.6 eV), where a significant hole doping corresponding to *E*_D_ ≈ +400 meV was reported^73^.

### Angle-resolved photoemission spectroscopy

Photoemission measurements were performed on cleaved FGT bulk single crystals and were conducted at NanoESCA beamline at Elettra, using the modified Focus GmbH NanoESCA momentum microscope. The sample was cooled using LHe and the temperature was stabilized at ≈40K. The light was incident at an angle 65° with respect to the surface normal, along the K-Γ-K’ direction. Supplementary Fig. S8b shows spectra collected at *ℏν* = 60 eV in sweep mode with an energy step size of 0.02 eV.

### Disentangling band orbital and chiral orbital Zeeman effects

We approximate the observed peak shifting behavior as function of magnetic field by the linear equation system

26 $${E}_{1}={g}_{1}{\mu }_{B}B+{E}_{1}^{0}=({g}_{z}^{{{\rm{CO}}}}-{g}_{z}^{{{\rm{BO}}}}){\mu }_{{{\rm{B}}}}B+{E}_{1}^{0}$$

27 $${E}_{2}={g}_{2}{\mu }_{B}B+{E}_{2}^{0}=({g}_{z}^{{{\rm{CO}}}}+{g}_{z}^{{{\rm{BO}}}}){\mu }_{{{\rm{B}}}}B+{E}_{2}^{0},$$

where *E*_*i*_(*B*) are the peak energies, with ${E}_{i}^{0}\equiv {E}_{i}(B=0)$, and ${g}_{z}^{{{\rm{CO}}}}$ and ${g}_{z}^{{{\rm{BO}}}}$ are the chiral and band orbital *g*-factors, respectively. Inserting the fitted slopes from Fig. 3c, *g*_1_*μ*_B_ = 1.8 meV/T and *g*_2_*μ*_B_ = 13.3 meV/T, results in

28 $${g}_{z}^{{{\rm{BO}}}}=\dfrac{{g}_{2}-{g}_{1}}{2}\approx 100$$

and $${g}_{z}^{{{\rm{CO}}}}\approx 130$$.

## Supplementary information

Peer Review File

Supplementary Information

## Data Availability

The data used in this study are available at Jülich Data^74^.

## References

[1] Armitage, N., Mele, E. & Vishwanath, A. Weyl and Dirac semimetals in three-dimensional solids. *Rev. Mod. Phys.* **90**, 015001 (2018).

[2] Wan, X., Turner, A. M., Vishwanath, A. & Savrasov, S. Y. Topological semimetal and Fermi-arc surface states in the electronic structure of pyrochlore iridates. *Phys. Rev. B* **83**, 205101 (2011).

[3] Tokura, Y., Yasuda, K. & Tsukazaki, A. Magnetic topological insulators. *Nat. Rev. Phys.* **1**, 126 (2019).

[4] Chen, B. et al. Magnetic properties of layered itinerant electron ferromagnet Fe_3_GeTe_2_. *J. Phys. Soc. Jpn.* **82**, 124711 (2013).

[5] Jang, S. W. et al. Origin of ferromagnetism and the effect of doping on Fe_3_GeTe_2_. *Nanoscale* **12**, 13501 (2020). 10.1039/c9nr10171c 32555905

[6] Zhu, J.-X. et al. Electronic correlation and magnetism in the ferromagnetic metal Fe_3_GeTe_2_. *Phys. Rev. B* **93**, 144404 (2016).

[7] Zhang, Y. et al. Emergence of Kondo lattice behavior in a van der Waals itinerant ferromagnet, Fe_3_GeTe_2_. *Sci. Adv.* **4**, eaao6791 (2018). 10.1126/sciadv.aao6791 PMC5770166 29349301

[8] Kim, T. J., Ryee, S. & Han, M. J. Fe_3_GeTe_2_: a site-differentiated Hund metal. *npj Comput. Mater.* **8**, 245 (2022).

[9] Kim, K. et al. Large anomalous Hall current induced by topological nodal lines in a ferromagnetic van der Waals semimetal. *Nat. Mater.* **17**, 794 (2018). 10.1038/s41563-018-0132-3 30013056

[10] Hanke, J.-P. et al. Role of Berry phase theory for describing orbital magnetism: From magnetic heterostructures to topological orbital ferromagnets. *Phys. Rev. B* **94**, 121114 (2016).

[11] Zhao, M. et al. Electric-field-driven negative differential conductance in 2D van der Waals ferromagnet Fe_3_GeTe_2_. *Nano Lett.* **21**, 9233 (2021). 10.1021/acs.nanolett.1c03123 34709835

[12] Zhao, M. et al. Kondo holes in the two-dimensional itinerant Ising ferromagnet Fe_3_GeTe_2_. *Nano Lett.* **21**, 6117 (2021). 10.1021/acs.nanolett.1c01661 34279960

[13] Srivastava, P. K. et al. Unusual Dzyaloshinskii-Moriya interaction in graphene/Fe_3_GeTe_2_ van der Waals heterostructure. *Small* **20**, 2402604 (2024). 10.1002/smll.202402604 38898739

[14] Deng, Y. et al. Gate-tunable room-temperature ferromagnetism in two-dimensional Fe_3_GeTe_2_. *Nature* **563**, 94 (2018). 10.1038/s41586-018-0626-9 30349002

[15] Wang, X. et al. Current-driven magnetization switching in a van der Waals ferromagnet Fe_3_GeTe_2_. *Sci. Adv.* **5**, eaaw8904 (2019). 10.1126/sciadv.aaw8904 PMC6707781 31467976

[16] Park, S. Y. et al. Controlling the magnetic anisotropy of the van der Waals ferromagnet Fe_3_GeTe_2_ through hole doping. *Nano Lett.* **20**, 95 (2019). 10.1021/acs.nanolett.9b03316 31752490

[17] Zhao, B., Karpiak, B., Hoque, A. M., Dhagat, P. & Dash, S. P. Strong perpendicular anisotropic ferromagnet Fe_3_GeTe_2_/graphene van der Waals heterostructure. *J. Phys. D: Appl. Phys.* **56**, 094001 (2023).

[18] Yin, J.-X. et al. Giant and anisotropic many-body spin–orbit tunability in a strongly correlated Kagome magnet. *Nature* **562**, 91 (2018). 10.1038/s41586-018-0502-7 30209398

[19] Lux, F. R., Freimuth, F., Blügel, S. & Mokrousov, Y. Engineering chiral and topological orbital magnetism of domain walls and skyrmions. *Commun. Phys.* **1**, 60 (2018).

[20] Grytsiuk, S. et al. Topological–chiral magnetic interactions driven by emergent orbital magnetism. *Nat. Commun.* **11**, 511 (2020). 10.1038/s41467-019-14030-3 PMC6981145 31980610

[21] Fei, Z. et al. Two-dimensional itinerant ferromagnetism in atomically thin Fe_3_GeTe_2_. *Nat. Mater.* **17**, 778 (2018). 10.1038/s41563-018-0149-7 30104669

[22] Zhang, Y. et al. Giant phonon-induced conductance in scanning tunnelling spectroscopy of gate-tunable graphene. *Nat. Phys.* **4**, 627 (2008).

[23] Wehling, T., Grigorenko, I., Lichtenstein, A. & Balatsky, A. Phonon-mediated tunneling into graphene. *Phys. Rev. Lett.* **101**, 216803 (2008). 10.1103/PhysRevLett.101.216803 19113437

[24] Decker, R. et al. Local electronic properties of graphene on a BN substrate via scanning tunneling microscopy. *Nano Lett.* **11**, 2291 (2011). 10.1021/nl2005115 21553853

[25] Natterer, F. D. et al. Strong asymmetric charge carrier dependence in inelastic electron tunneling spectroscopy of graphene phonons. *Phys. Rev. Lett.* **114**, 245502 (2015). 10.1103/PhysRevLett.114.245502 26196985

[26] Qiu, Z. et al. Visualizing atomic structure and magnetism of 2D magnetic insulators via tunneling through graphene. *Nat. Commun.* **12**, 1 (2021). 10.1038/s41467-020-20376-w PMC7782518 33397960

[27] Sun, L. et al. Determining spin-orbit coupling in graphene by quasiparticle interference imaging. *Nat. Commun.* **14**, 3771 (2023). 10.1038/s41467-023-39453-x PMC10290717 37355633

[28] Li, H. et al. Manipulation of Dirac band curvature and momentum-dependent *g*-factor in a Kagome magnet. *Nat. Phys.* **18**, 644 (2022).

[29] Li, H. et al. Spin Berry curvature-enhanced orbital Zeeman effect in a Kagome metal. *Nat. Phys.* **20**, 1103 (2024).

[30] Jo, D. et al. Weak ferromagnetism in altermagnets from alternating *g*-Tensor anisotropy. *Phys. Rev. Lett.* **134**, 196703 (2025). 10.1103/PhysRevLett.134.196703 40446241

[31] Mühlbauer, S. et al. Skyrmion lattice in a chiral magnet. *Science* **323**, 915 (2009). 10.1126/science.1166767 19213914

[32] Li, D., Haldar, S., Drevelow, T. & Heinze, S. Tuning the magnetic interactions in van der Waals Fe_3_GeTe_2_ heterostructures: a comparative study of ab initio methods. *Phys. Rev. B* **107**, 104428 (2023).

[33] Shindou, R. & Nagaosa, N. Orbital ferromagnetism and anomalous Hall effect in antiferromagnets on the distorted fcc lattice. *Phys. Rev. Lett.* **87**, 116801 (2001). 10.1103/PhysRevLett.87.116801 11531542

[34] Tatara, G. & Garcia, N. Quantum toys for quantum computing: Persistent currents controlled by the spin Josephson effect. *Phys. Rev. Lett.* **91**, 076806 (2003). 10.1103/PhysRevLett.91.076806 12935044

[35] Bulaevskii, L., Batista, C., Mostovoy, M. & Khomskii, D. Electronic orbital currents and polarization in Mott insulators. *Phys. Rev. B* **78**, 024402 (2008).

[36] dos Santos Dias, M., Bouaziz, J., Bouhassoune, M., Blügel, S. & Lounis, S. Chirality-driven orbital magnetic moments as a new probe for topological magnetic structures. *Nat. Commun.* **7**, 13613 (2016). 10.1038/ncomms13613 PMC5187428 27995909

[37] Niu, C. et al. Mixed topological semimetals driven by orbital complexity in two-dimensional ferromagnets. *Nat. Commun.* **10**, 3179 (2019). 10.1038/s41467-019-10930-6 PMC6639329 31320628

[38] Ko, E. Hybridized bands and stacking-dependent band edges in ferromagnetic Fe_3_GeTe_2_/CrGeTe_3_ moiré heterobilayer. *Sci. Rep.* **12**, 1 (2022). 10.1038/s41598-022-08785-x PMC8948266 35332178

[39] Yu, Y.-J. et al. Tuning the graphene work function by electric field effect. *Nano Lett.* **9**, 3430 (2009). 10.1021/nl901572a 19719145

[40] Huang, S. et al. Giant magnetoresistance induced by spin-dependent orbital coupling in Fe_3_GeTe_2_/graphene heterostructures. *Nat. Commun.* **16**, 2866 (2025). 10.1038/s41467-025-58224-4 PMC11933411 40128534

[41] Kim, K. S. et al. Visualizing atomic-scale negative differential resistance in bilayer graphene. *Phys. Rev. Lett.* **110**, 036804 (2013). 10.1103/PhysRevLett.110.036804 23373943

[42] Luican-Mayer, A. et al. Negative differential resistance observed on the charge density wave of a transition metal dichalcogenide. *Nanoscale* **11**, 22351 (2019). 10.1039/c9nr07857f 31728463

[43] Yin, L.-J. et al. Imaging of nearly flat band induced atomic-scale negative differential conductivity in ABC-stacked trilayer graphene. *Phys. Rev. B* **102**, 241403 (2020).

[44] García-Díez, M., Beidenkopf, H., Robredo, I. & Vergniory, M. G. Origins of the anomalous Hall conductivity in the symmetry enforced Fe_3_GeTe_2_ nodal-line ferromagnet. *J. Phys. Mater.* **8**, 035012 (2025).

[45] Sales, B. C. et al. Electronic, magnetic, and thermodynamic properties of the Kagome layer compound FeSn. *Phys. Rev. Mater.* **3**, 114203 (2019).

[46] Yin, J.-X. et al. Negative flat band magnetism in a spin–orbit-coupled correlated Kagome magnet. *Nat. Phys.* **15**, 443 (2019).

[47] Xing, Y. et al. Localized spin-orbit polaron in magnetic Weyl semimetal Co_3_Sn_2_S_2_. *Nat. Commun.* **11**, 5613 (2020). 10.1038/s41467-020-19440-2 PMC7644724 33154384

[48] Ren, Z. et al. Plethora of tunable Weyl fermions in Kagome magnet Fe_3_Sn_2_ thin films. *npj Quantum Mater.* **7**, 109 (2022).

[49] Taychatanapat, T. & Jarillo-Herrero, P. Electronic transport in dual-gated bilayer graphene at large displacement fields. *Phys. Rev. Lett.* **105**, 166601 (2010). 10.1103/PhysRevLett.105.166601 21230989

[50] Uwanno, T., Taniguchi, T., Watanabe, K. & Nagashio, K. Electrically inert h-bn/bilayer graphene interface in all-two-dimensional heterostructure field effect transistors. *ACS Appl. Mater. Interfaces* **10**, 28780 (2018). 10.1021/acsami.8b08959 30080037

[51] Chen, H. et al. Gate controlled valley polarizer in bilayer graphene. *Nat. Commun.* **11**, 1202 (2020). 10.1038/s41467-020-15117-y PMC7058031 32139694

[52] Wortmann, D. et al. FLEUR, Code available at 10.5281/zenodo.7576163 (2023).

[53] Wimmer, E., Krakauer, H., Weinert, M. & Freeman, A. J. Full-potential self-consistent linearized-augmented-plane-wave method for calculating the electronic structure of molecules and surfaces: O_2_ molecule. *Phys. Rev. B* **24**, 864 (1981).

[54] Perdew, J. P., Burke, K. & Ernzerhof, M. Generalized gradient approximation made simple. *Phys. Rev. Lett.* **77**, 3865 (1996). 10.1103/PhysRevLett.77.3865 10062328

[55] Deiseroth, H.-J., Aleksandrov, K., Reiner, C., Kienle, L. & Kremer, R. K. Fe_3_GeTe_2_ and Ni_3_GeTe_2_ - Two new layered transition-metal compounds: crystal structures, HRTEM investigations, and magnetic and electrical properties. *Eur. J. Inorg. Chem.* **8**, 1561 (2006).

[56] Wang, X., Yates, J. R., Souza, I. & Vanderbilt, D. Ab initio calculation of the anomalous Hall conductivity by Wannier interpolation. *Phys. Rev. B* **74**, 195118 (2006).

[57] Pizzi, G. et al. Wannier90 as a community code: new features and applications. *J. Phys. Condens. Matter* **32**, 165902 (2020). 10.1088/1361-648X/ab51ff 31658458

[58] Freimuth, F., Mokrousov, Y., Wortmann, D., Heinze, S. & Blügel, S. Maximally localized Wannier functions within the FLAPW formalism. *Phys. Rev. B* **78**, 035120 (2008).

[59] Lopez, M., Vanderbilt, D., Thonhauser, T. & Souza, I. Wannier-based calculation of the orbital magnetization in crystals. *Phys. Rev. B* **85**, 014435 (2012).

[60] Saunderson, T. G., Go, D., Blügel, S., Kläui, M. & Mokrousov, Y. Hidden interplay of current-induced spin and orbital torques in bulk Fe_3_GeTe_2_. *Phys. Rev. Res.* **4**, L042022 (2022).

[61] Wakafuji, Y. et al. 3D manipulation of 2D materials using microdome polymer. *Nano Lett.* **20**, 2486 (2020). 10.1021/acs.nanolett.9b05228 32155082

[62] Nečas, D. & Klapetek, P. Gwyddion: an open-source software for SPM data analysis. *Cent. Eur. J. Phys.* **10**, 181 (2012).

[63] Brun, C. et al. Dynamical Coulomb blockade observed in nanosized electrical contacts. *Phys. Rev. Lett.* **108**, 126802 (2012). 10.1103/PhysRevLett.108.126802 22540609

[64] Andrei, E. Y., Li, G. & Du, X. Electronic properties of graphene: a perspective from scanning tunneling microscopy and magnetotransport. *Rep. Prog. Phys.* **75**, 056501 (2012). 10.1088/0034-4885/75/5/056501 22790587

[65] Lee, J. et al. Imaging electrostatically confined dirac fermions in graphene quantum dots. *Nat. Phys.* **12**, 1032 (2016).

[66] Ge, Z. et al. Giant orbital magnetic moments and paramagnetic shift in artificial relativistic atoms and molecules. *Nat. Nanotechnol.* **18**, 250 (2023). 10.1038/s41565-023-01327-0 36879123

[67] Lee, Y. et al. Tunable valley splitting due to topological orbital magnetic moment in bilayer graphene quantum point contacts. *Phys. Rev. Lett.* **124**, 126802 (2020). 10.1103/PhysRevLett.124.126802 32281833

[68] Ge, Z. et al. Control of giant topological magnetic moment and valley splitting in trilayer graphene. *Phys. Rev. Lett.* **127**, 136402 (2021). 10.1103/PhysRevLett.127.136402 34623864

[69] Black, C. T. & Welser, J. J. Electric-field penetration into metals: consequences for high-dielectric-constant capacitors. *IEEE Trans. Electron Devices* **46**, 776 (1999).

[70] Ambrosetti, A. & Silvestrelli, P. L. Faraday-like screening by two-dimensional nanomaterials: a scale-dependent tunable effect. *J. Phys. Chem. Lett.* **10**, 2044 (2019). 10.1021/acs.jpclett.9b00860 30964300

[71] Lüpke, F. et al. In situ disentangling surface state transport channels of a topological insulator thin film by gating. *npj Quantum Mater.* **3**, 46 (2018).

[72] Xia, J., Chen, F., Li, J. & Tao, N. Measurement of the quantum capacitance of graphene. *Nat. Nanotechnol.* **4**, 505 (2009). 10.1038/nnano.2009.177 19662012

[73] Chen, Y. et al. Visualizing the anomalous charge density wave states in graphene/NbSe_2_ heterostructures. *Adv. Mater.* **32**, 2003746 (2020). 10.1002/adma.202003746 33002238

[74] Wichmann, T. et al. Jülich Data, 10.26165/JUELICH-DATA/CNFW54 (2026).

[75] Momma, K. & Izumi, F. VESTA 3 for three-dimensional visualization of crystal, volumetric and morphology data. *Appl. Crystallogr.* **44**, 1272 (2011).

