# Supplementary material for: Giant orbital Zeeman effects in a magnetic topological van der Waals interphase
Source: Nat Commun. 2026 Oct 23;17:11076. doi: 10.1038/s41467-026-78016-8 (PMC13694696; doi:10.1038/s41467-026-78016-8)
Supplement: Supplementary file 1 [file 41467_2026_78016_MOESM1_ESM.pdf]

nature portfolio

Peer Review File

Giant orbital Zeeman effects in a magnetic topological van der Waals interphase

Corresponding Author: Dr Felix Luepke

Parts of this Peer Review File have been redacted as indicated to remove third-party material.

This file contains all reviewer reports in order by version, followed by all author rebuttals in order by version.

Version 0:

Reviewer comments:

Reviewer #1

(Remarks to the Author)
Report on manuscript " Giant orbital Zeeman effects in a magnetic topological van der Waals interface" by Wichmann et al. This manuscript describes an energy splitting in tunneling spectra on graphene on FGT where the energy splitting exhibits a vary with applied magnetic field. The authors interpret this as a giant orbital effect arising from a combination of band orbital moments and chiral orbital moments. The splitting is also sensitive to applied electric fields, which is a novel finding. The authors have made a good case for the explanation with the support of theoretical calculations. Overall, the paper is novel in characterizing an electrically controlled interphase magnetism.

Specific comments on the manuscript:
1. The authors observe a soft gap in the tunnelling spectrum which they interpret as a phonon gap providing the needed inelastic connection to the K/K' points. However, an examination of the graphitic phonon spectra shows that there are no K phonons at this energy, as seen in Ref. L. Vitali, M. A. Schneider, K. Kern, L. Wirtz, and A. Rubio, Phys. Rev. B 69, 121414 (2004). Other authors observe this gap with more or less definition and have also interpreted as a dynamical Coulomb suppression gap. Maybe the authors can clarify this point.

2. All the results stem from two relatively very small features compared to the main spectra intensity in the overall tunnel spectra (Fig. 3a). It seems somewhat a stretch to make such a detailed story for these two small features, since they are not very prominent. Why are they not more prominent compared to the rest of the overall spectra?

3. Already at 0.5 T graphene should display magnetic Landau levels. Is it possible these peaks are graphene LLs that are misinterpreted? The LLs that are closest to Ef will have the largest intensity due to lifetimes effects, and therefore the other LLs could be washed out due to disorder. Could these two peaks be N=1 or 2 LLs, or N=2 or 3, for example. If not, why are the graphene LLs not observed then.

Reviewer #2

(Remarks to the Author)
The authors present a combined experimental STM and theoretical study that investigates an interface state in a graphene/Fe_3 GeTe_2 heterostructure. Overall, the authors found that this state exhibits a notable magneto-electric effect. First the authors demonstrate the local density of states of Fe_3 GeTe_2 (FGT) can be accessed directly within the low tip-sample bias tunneling regime. The authors attribute this to the presence of an inelastic tunneling "gap" in graphene due to its zero density of states at zero momenta(gamma point). By mostly restricting themselves to this bias range and then applying a perpendicular magnetic field the authors find two peaks that shift linearly with increasing magnetic field. The authors then keep the applied magnetic field constant and change the tip-sample distance and find that this also causes a shift in the peaks observed at zero magnetic field. They interpret this latter effect as a change in the applied electric field due to the work function mismatch between graphene and the STM tip. Essentially, the changing tip-sample distance enables modulation of the applied electric field. The authors ascribe the response of the peaks to changing magnetic and electric fields to a magneto electric effect of a magnetic interface state. Such a state is attributed to the coupling between orbital and spin magnetic moments hosted by the FGT surface. The orbital magnetic moment is topological in origin and is calculated with

DFT. The associated g-factor for the observed magnetic moments is determined from the magnetic and electric field dependent data.

Electrically controlled magnetism is an alluring capability and is pursued through a variety of approaches and material platforms in condensed matter physics. The authors claim they have discovered a new magneto-electric effect via STM measurements arising from the interplay of magnetism and topology in a graphene/Fe_3 GeTe_2 heterostructure. The experimental results are interesting however it is unclear whether the data shown here can be solely attributed to magnetic moments at the interface of the heterostructure. Additionally, even if the authors' interpretation was correct gate tunable orbital magnetic moments have been realized before in graphene. Because of these weaknesses I cannot recommend publication of this work in Nature Communications in its current stage. Below I elaborate on my criticisms and can consider this manuscript again if the authors address my criticisms.

1) The authors claim they can leverage the inelastic tunneling "gap" in graphene to access FGT directly. However, their DFT bandstructure calculation of FGT shows that the gamma point also has zero states like graphene and so one would expect FGT to display zero dI/dV signal at low voltage biases as well. Considering these points the authors need to explain how they can bypass graphene but still access FGT even though both have zero states at the gamma point.

2) Elastic tunneling into graphene is possible and would serve as an important check on the nature of the features observed by the authors. Since elastic tunneling would probe the graphene states the features related to the FGT should be absent. The authors should comment on the nature of their peak features for an elastic tunneling response.

3) Increasing the current setpoint will enhance the elastic tunneling channel, see J. Lee et al Nature Physics 2016. For measurements where the current setpoint is increased the authors could also be enhancing the elastic tunneling channel into graphene. The presence of peaks could then be attributed to quantum dot (QD) states from the tip gating effect, see Y. Zhao et al. Science 2015. These states also have a strong response to magnetic and electric fields. The authors need to rule out that the peaks seen in their experiments are not related to QD states from the STM tip gating effect.

4) The tip gating electric field is much larger than the gate electric field from conventional device architectures. This makes the authors finding less broadly applicable. The authors should address this point.

5) Magnetic and electric field tunable orbital magnetic moments have been seen before see works by Y. Lee et al. Physical Review Letters 2020, Z. Ge et al. Physical Review Letters 2021, and Z. Ge et al., Nature Nanotechnology 2023. The authors must include these prior works in their discussions and compare their findings with these prior works.

Version 1:

Reviewer comments:

Reviewer #1

(Remarks to the Author)
The authors have answered my comments sufficiently and I recommend publication in NC.

Reviewer #2

(Remarks to the Author)
I thank the authors for their thoughtful responses to my questions and criticisms. They have fully addressed all of my points. In particular, the additional discussion that compares the reciprocal unit cells of graphene and FGT is very helpful for understanding the authors' tunneling experiments. I now recommend this manuscript for publication in Nature Communications.

Open Access This Peer Review File is licensed under a Creative Commons Attribution 4.0 International License, which permits use, sharing, adaptation, distribution and reproduction in any medium or format, as long as you give appropriate credit to the original author(s) and the source, provide a link to the Creative Commons license, and indicate if changes were made.
In cases where reviewers are anonymous, credit should be given to 'Anonymous Referee' and the source.
The images or other third party material in this Peer Review File are included in the article's Creative Commons license, unless indicated otherwise in a credit line to the material. If material is not included in the article's Creative Commons license and your intended use is not permitted by statutory regulation or exceeds the permitted use, you will need to obtain permission directly from the copyright holder.
To view a copy of this license, visit https://creativecommons.org/licenses/by/4.0/

Rebuttal – NCOMMS-25-103599-T

June 17, 2026

Answer to the Reviewer #1:

Report on manuscript " Giant orbital Zeeman effects in a magnetic topological van der Waals interface" by Wichmann et al. This manuscript describes an energy splitting in tunneling spectra on graphene on FGT where the energy splitting exhibits a vary with applied magnetic field. The authors interpret this as a giant orbital effect arising from a combination of band orbital moments and chiral orbital moments. The splitting is also sensitive to applied electric fields, which is a novel finding. The authors have made a good case for the explanation with the support of theoretical calculations. Overall, the paper is novel in characterizing an electrically controlled interphase magnetism.

Specific comments on the manuscript:
1. The authors observe a soft gap in the tunnelling spectrum which they interpret as a phonon gap providing the needed inelastic connection to the K/K' points. However, an examination of the graphitic phonon spectra shows that there are no K phonons at this energy, as seen in Ref. L. Vitali, M. A. Schneider, K. Kern, L. Wirtz, and A. Rubio, Phys. Rev. B 69, 121414 (2004). Other authors observe this gap with more or less definition and have also interpreted as a dynamical Coulomb suppression gap. Maybe the authors can clarify this point.

Reply: We thank the reviewer for their positive assessment of our work and for bringing up this important point. Regarding the origin of the tunneling gap (phonon-assisted inelastic tunneling vs. dynamical Coulomb blockade), our results allow us to rule out the latter scenario: In our investigated sample system the graphene is in direct contact to an extended metallic substrate (the FGT), which strongly suppresses any dynamical Coulomb blockade effects compared to graphene on insulating substrates [Phys. Rev. Lett. 108, 126802 (2012)]. Yet, the observed gap remains almost unchanged compared to graphene on hBN, SiO_2 and SiC substrates. We thus conclude that the dynamical Coulomb blockade is not relevant here and follow the interpretation of a substrate-independent phonon-assisted inelastic tunneling mechanism: Our observed onset energy of the inelastic tunnelling closely corresponds to the lowest lying phonon mode at the K point, in agreement with theoretical calculations, such as the one in the mentioned reference [Phys. Rev. B 69, 121414 (2004)], see Fig. R1 below. The fact that the inelastic gap is visible in some experimental spectra found in the literature and in others not, in our experience is the result of different tunneling tip properties. A possible explanation is that a sharp tunneling tip has a rather broad distribution of states in k-space, whereas for a blunt tip states are more localized at $k = 0$, due to real-space/momentum-space conjugacy. In this case, with a sharp tip the graphene K points are more directly accessible via elastic tunneling channels, whereas with a blunt tip the elastic tunneling is suppressed and inelastic tunneling channels become more dominant.

[Figure Redacted]

Figure R1: Graphite phonon spectrum from Phys. Rev. B 69, 121414 (2004). The red arrow highlights the lowest -lying K-point phonon, the energy of which corresponds to the observed inelastic excitation.

We have added this additional information to the Methods section of the manuscript and included the mentioned reference there:

"We further note that, because the graphene is in direct contact to the extended metallic FGT flake, dynamical Coulomb blockade effects are strongly suppressed in our experiments [62] and thus cannot explain our observed gap features. This argument is supported by the fact that the observed inelastic gaps are almost identical compared to graphene on insulating substrates [26, 27, 30]."

2. All the results stem from two relatively very small features compared to the main spectra intensity in the overall tunnel spectra (Fig. 3a). It seems somewhat a stretch to make such a detailed story for these two small features, since they are not very prominent. Why are they not more prominent compared to the rest of the overall spectra?

Reply: We thank the reviewer for this thoughtful comment. Indeed the features close to EF have smaller intensities than those at higher energies. On the one hand, this is an intrinsic property of tunneling junctions where the tunneling barrier takes on a trapezoidal shape when a bias is applied, resulting in a non-linear dependence of the tunneling probability as the voltage is increased (see below). In graphene, the intensity difference between states at lower and higher tunneling bias is further enhanced by the inelastic tunneling channels that become available at $|eV| > E_{\mathrm{ph}}$. Since the tunneling current to the graphene for $|eV| < E_{\mathrm{ph}}$ is small, tunneling from the FGT dominates. The FGT, however, is further away from the tip than the graphene and thus has lower tunneling probability. Yet, because of the significantly higher density of states of the FGT compared to the graphene, it dominates the tunneling for $|eV| < E_{\mathrm{ph}}$ – see the detailed analysis in the reply to the second reviewer. In summary, our experiment shows a cascade of tunneling intensities stemming from the different elastic and inelastic tunneling channels, which we are able to distinguish and interpret separately. In particular, the interpretation of FGT features inside the inelastic gap of the graphene is thus not compromised by the large tunneling intensity of the latter at $|eV| \geq E_{\mathrm{ph}}$. We have included a detailed discussion of this in the Methods section of the manuscript:

"The trapezoidal shape of the biased tunneling barrier in the WKB-approximation results in a non-linear dependence of the tunneling conductance as the voltage is increased:

$$dI/dV \propto \rho\, e^{-2\kappa(E)z}, \tag{9}$$

with the density of states ρ, the decay constant

$$\kappa(E) = \sqrt{\frac{2m}{\hbar^2}(\Phi_{\mathrm{eff}} - E) + k_{\parallel}^2}, \tag{10}$$

the effective barrier height

$$\Phi_{\text{eff}} = \frac{\Phi_{\text{tip}} + \Phi_{\text{sample}}}{2} \tag{11}$$

for tunneling bias $E = eV_{\text{s}} \ll \Phi_{\text{eff}}$, and the momentum in the surface plane $k_{\|}$. As a result, states close to E_{F} tend to have a smaller dI/dV intensity than those at higher bias. In graphene, the intensity difference between states at lower and higher tunneling bias is further enhanced by the inelastic tunneling channels that become available at $|eV| > E_{\text{ph}}$, for which the tunneling probability is significantly increased [27]. Inside this inelastic gap, the elastic tunneling current to the graphene is small, such that tunneling from the FGT dominates: Although the FGT is further away from the tip than the graphene and thus has lower tunneling probability (Eq. 9), because of its significantly higher density of states compared to the graphene, it dominates the tunneling for $|eV| < E_{\text{ph}}$.

3. Already at 0.5 T graphene should display magnetic Landau levels. Is it possible these peaks are graphene LLs that are misinterpreted? The LLs that are closest to Ef will have the largest intensity due to lifetimes effects, and therefore the other LLs could be washed out due to disorder. Could these two peaks be N=1 or 2 LLs, or N=2 or 3, for example. If not, why are the graphene LLs not observed then.

Reply: While in ultra-clean freestanding graphene, Landau levels can be observed at 0.5 T, in our present study the graphene is coupled to a disordered substrate, the FGT. In this scenario, Landau levels typically are only realized at much higher fields because the graphene carrier mean free path is significantly reduced and as a result, the magnetic field required to create coherently closed electron trajectories (cyclotron orbits) increases. This is already the case when graphene is coupled to substrates much less disordered than the FGT [e.g., Andrei et al., Rep. Prog. Phys. 75, 056501 (2012)]. Our interpretation is further supported by the fact that we already observe the two-peak dI/dV features at $0\,\text{T}$ and that the dependence on the observed peak splitting is linear in the magnetic field $E \propto B$ (Fig. 3c), i.e. it does not follow the typical Landau level dispersion of graphene: $E_n = E_{\text{D}} \cdot \text{sng}(n)\sqrt{2ehvB|n|} \propto \sqrt{B}$. Lastly, when tuning the graphene doping level, the peak features do not follow the observed shift of the Dirac cone (Fig. 4). We take this as strong evidence that the peak features do not originate in the graphene, and must come from the FGT instead. We have included this discussion in the manuscript:

"Lastly, the combined observation of a finite peak splitting at zero field followed by an approximately linear shift as function of magnetic field effectively rules out Landau level formation in the graphene as a potential origin of the peak features, since they would follow a square-root dependence $E \propto \sqrt{B}$ in the absence of lateral confinement [63]. This is further supported by the fact that the setpoint-dependent peak shifts (Fig. 4) are clearly different from the change in the graphene doping level, which we take as the most direct proof that they originate in the FGT and not the graphene."

Answer to the Reviewer #2:

The authors present a combined experimental STM and theoretical study that investigates an interface state in a graphene/Fe_3 GeTe_2 heterostructure. Overall, the authors found that this state exhibits a notable magneto-electric effect. First the authors demonstrate the local density of states of Fe_3 GeTe_2 (FGT) can be accessed directly within the low tip-sample bias tunneling regime. The authors attribute this to the presence of an inelastic tunneling "gap" in graphene due to its zero density of states at zero momenta(gamma point). By mostly restricting themselves to this bias range and then applying a perpendicular magnetic field the authors find two peaks that shift linearly with increasing magnetic field. The authors then keep the applied magnetic field constant and change the tip-sample distance and find that this also causes a shift in the peaks observed at zero magnetic field. They interpret this latter effect as a change in the applied electric field due to the work function mismatch between graphene and the STM tip. Essentially, the changing tip-sample distance enables modulation of the applied electric field. The authors ascribe the response of the peaks to changing magnetic and electric fields to a magneto electric effect of a magnetic interface state. Such a state is attributed to the coupling between orbital and spin magnetic moments hosted by the FGT surface. The orbital magnetic moment is topological in origin and is calculated with DFT. The associated g-factor for the observed magnetic moments is determined from the magnetic and electric field dependent data.

Electrically controlled magnetism is an alluring capability and is pursued through a variety of approaches and material platforms in condensed matter physics. The authors claim they have discovered a new magneto-electric effect via STM measurements arising from the interplay of magnetism and topology in a graphene/Fe_3 GeTe_2 heterostructure. The experimental results are interesting however it is unclear whether the data shown here can be solely attributed to magnetic moments at the interface of the heterostructure. Additionally, even if the authors' interpretation was correct gate tunable orbital magnetic moments have been realized before in graphene. Because of these weaknesses I cannot recommend publication of this work in Nature Communications in its current stage. Below I elaborate on my criticisms and can consider this manuscript again if the authors address my criticisms.

1) The authors claim they can leverage the inelastic tunneling "gap" in graphene to access FGT directly. However, their DFT bandstructure calculation of FGT shows that the gamma point also has zero states like graphene and so one would expect FGT to display zero dI/dV signal at low voltage biases as well. Considering these points the authors need to explain how they can bypass graphene but still access FGT even though both have zero states at the gamma point.

> **Reply:** We thank the reviewer for their constructive criticism, which we address below. Indeed, FGT, like graphene, has no states exactly at $k = \Gamma$ and E_F. However, tunneling not only occurs at $k = \Gamma$, but the tunneling probability rather follows an exponential dependence outlined in Eqs. (9-11) above. Below, we analyze the elastic tunneling current contributions in detail and find that the FGT dominates the tunneling inside the inelastic gap: Because the FGT's reciprocal unit cell is significantly smaller than that of graphene, its states are closer to $k = \Gamma$ than the graphene Dirac states (see Fig. R2). Then, according to Eq. 10, the tunneling decay constant to the FGT states is smaller than that of the graphene. This effect is further enhanced by the lower work function of FGT compared to graphene, which further reduces the FGT decay constant. This smaller FGT decay constant is counteracted by the fact that the FGT is further away from the tip ($z \approx 13.34$ Å) than the graphene ($z \approx 10$ Å). However, the tunneling current also scales with the density of states, which for FGT is significantly higher than for graphene, because the Fe $3d$ bands near K are much flatter than the graphene π-bands (take note of the different energy scales in Fig. R2b). We have added a corresponding quantitative analysis to the Methods section of the manuscript, which concludes that the FGT dominates the tunneling inside the inelastic gap:
>
> *"We estimate the elastic tunneling probability at the Fermi energy ($E = 0$) from Eqs. (9-11). For graphene ($k_\parallel = 1.7$ Å^{-1} and $z = h = 10$ Å), it results $e^{-2\kappa h} \approx 3 \cdot 10^{-18}$. For FGT on the other hand ($k_\parallel = 1.05$ Å^{-1}*

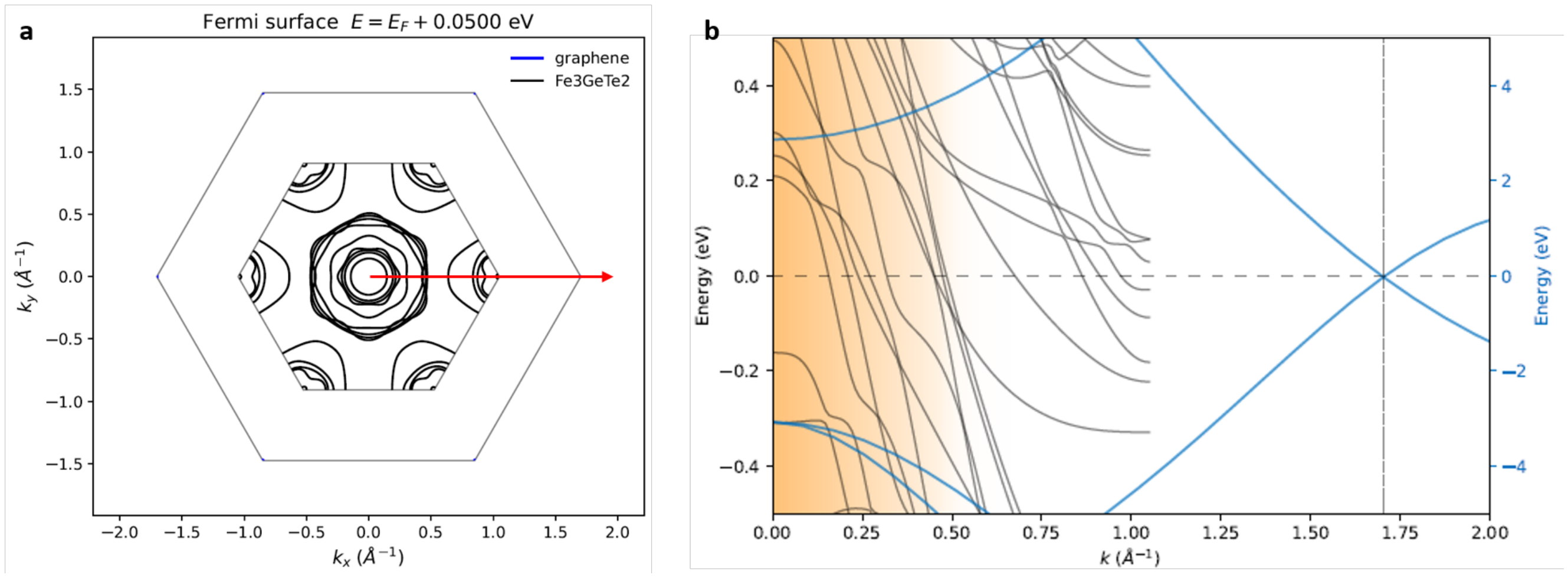

Figure R2: **(a)** Combined Fermi surfaces of FGT and graphene $50\,\mathrm{meV}$ above the Dirac point energy. **(b)** Band structure of FGT and graphene plotted on the same in-plane momentum axis, from Γ to K, corresponding to the red arrow in panel a. Note the two different y-axis energy scales (grey: FGT, blue: graphene). The orange background corresponds to the in-plane momentum dependence of the tunneling probability to the graphene according to Eq. 10, plotted on a logarithmic scale.

> *and $z = h + d = 13.34\,Å$), it results $e^{-2\kappa(h+d)} \approx 5 \cdot 10^{-18}$. Thus, the theoretical elastic tunneling probability to the K-point states in the FGT is larger than in the graphene. In addition, since FGT has a significantly higher density of states than graphene, because the Fe 3d bands near K are flatter than the graphene π-bands, we conclude that the tunneling inside the inelastic gap is dominated by the FGT."*

2) Elastic tunneling into graphene is possible and would serve as an important check on the nature of the features observed by the authors. Since elastic tunneling would probe the graphene states the features related to the FGT should be absent. The authors should comment on the nature of their peak features for an elastic tunneling response.

> **Reply:** When inelastic tunneling channels are accessible, they dominate the tunneling into graphene: the differential conductance of the inelastic channels is about an order of magnitude larger compared to the elastic channels [Nature Physics 4, 627 (2008)]. Consequently, only within the inelastic gap can the elastic tunneling into graphene be probed without being masked by the inelastic contributions. In our heterostructure, however, the differential conductance inside the inelastic gap is dominated by the FGT – see point above – and thus the elastic tunneling spectrum of the graphene cannot be isolated in our sample. Yet, the elastic graphene spectrum has been studied in detail on insulating substrates, e.g. [Nature Physics 4, 627 (2008)], which due to the weak interfacial van der Waals interactions should be directly comparable. In such experiments, no peaks have been observed that showed a similar B-field behavior, unless the graphene states were laterally confined – see discussion below.

3) Increasing the current setpoint will enhance the elastic tunneling channel, see J. Lee et al Nature Physics 2016. For measurements where the current setpoint is increased the authors could also be enhancing the elastic tunneling channel into graphene. The presence of peaks could then be attributed to quantum dot (QD) states from the tip gating effect, see Y. Zhao et al. Science 2015. These states also have a strong response to magnetic and electric fields. The authors need to rule out that the peaks seen in their experiments are not related to QD states from the STM tip gating effect.

> **Reply:** Increasing the current setpoint will increase the elastic tunneling probability in both, the graphene and the FGT: Bringing the tip closer to the sample by $1\,Å$ according to Eqs. 9-11, increases the tunneling probability for both, the graphene and the FGT K-point states, by almost two order of magnitude, to

$1.5 \cdot 10^{-16}$ and $0.95 \cdot 10^{-16}$, respectively. Thus, in the range of experimental setpoints 0.1-1 nA used in Fig. 4, only negligible changes in the ratio of the different tunneling channels occur, and we still expect the FGT to dominate the tunneling inside the inelastic gap, due to its much higher density of states.

To realize a graphene quantum dot requires a potential well in which charge carriers are confined. In our heterostructure, the graphene is coupled to the metallic FGT, which renders it n-doped everywhere, such that we expect confinement to be weak. Furthermore, our observations are not consistent with conventional quantum-dot (QD) bound states or whispering gallery modes (WGMs). Such states typically give rise to a series of peaks in the density of states corresponding to bound states with increasing (angular) momentum quantum numbers. In contrast, our heterostructure exhibits only two well-isolated peak features within the inelastic gap. These features instead are phenomenologically much more similar to tunable orbital moments of a gapped graphene Dirac cone reported in [Z. Ge et al., PRL 127, 136402 (2021)]. In that work, lateral carrier confinement in electrostatically defined potential wells lifts the graphene's inversion symmetry and gives rise to orbital magnetic moments, which couple to external electric and magnetic fields. In contrast to this work however, in our heterostructure, both, inversion and time-reversal symmetry are already broken, such that we do not require lateral confinement to realize field tunable orbital moments. Instead, we observe the orbital moments of the gapped FGT nodal line, which dominates the tunneling. This interpretation is supported by the fact that the states intrinsic to the graphene shift according to the graphene doping level in [Z. Ge et al. PRL 127, 136402 (2021)], whereas the peaks in our experiments do not: In Fig. 4a, we demonstrate that the graphene doping level can be tuned by $\Delta E_{\mathrm{D}} \sim 15\,\mathrm{meV}$ by changing the tip-sample distance. However, the peaks inside the inelastic gap only shift asymmetrically by $\lesssim 1\,\mathrm{meV}$, which points to their origin not in the graphene, but in the FGT. This interpretation is consistent with our argument above, that tunneling inside the inelastic gap is dominated by the FGT. Lastly, we rule out the formation of a quantum dot in the FGT itself, because of its metallic nature and resulting weak charge confinement. We have added this discussion to the Methods section of the manuscript:

"The combined observation of a finite peak splitting at zero field followed by an approximately linear shift as function of magnetic field effectively rules out Landau level formation in the graphene as a potential origin of the peak features, since they would follow a square-root dependence $E \propto \sqrt{B}$ in the absence of lateral confinement [66]. We expect lateral confinement to be weak in our heterostructure, because the graphene is coupled to the metallic FGT, which renders it n-doped everywhere. Furthermore, our observations are not consistent with conventional quantum-dot (QD) bound states or whispering gallery modes (WGMs): Such states typically give rise to a series of peaks in the density of states corresponding to bound states with increasing (angular) momentum quantum numbers [64, 65]. In contrast, our heterostructure exhibits only two well-isolated peak features within the inelastic gap. These features instead are phenomenologically much more similar to tunable orbital moments of a gapped graphene Dirac cone [66, 67]. In those works, lateral carrier confinement in electrostatically defined potential wells lifts the graphene's inversion symmetry and gives rise to orbital magnetic moments, which couple to external electric and magnetic fields. In contrast to this work however, in our heterostructure, both, inversion and time-reversal symmetry are already broken, such that we do not require lateral confinement to realize field tunable orbital moments. Instead, we observe the orbital moments of the gapped FGT nodal line, which dominates the tunneling. This interpretation is supported by the fact that the states intrinsic to the graphene shift according to the graphene doping level [67], whereas the peaks in our experiments do not: In Fig. 4a, we demonstrate that the graphene doping level can be tuned by $\Delta E_{\mathrm{D}} \sim 15\,\mathrm{meV}$ by changing the tip-sample distance. However, the peaks inside the inelastic gap only shift asymmetrically by $\lesssim 1\,\mathrm{meV}$, which points to their origin not in the graphene, but in the FGT. This interpretation is consistent with our argument above, that tunneling inside the inelastic gap is dominated by the FGT. Lastly, we rule out the formation of a quantum dot in the FGT itself, because of its metallic nature."

4) The tip gating electric field is much larger than the gate electric field from conventional device architectures. This makes the authors finding less broadly applicable. The authors should address this point.

Reply: This is incorrect: State-of-the-art dual-gated graphene transport devices readily achieve electric fields $> 1\,\mathrm{GV/m}$ [Uwanno et al. ACS Appl. Mater. Interfaces 10, 28780 (2018), Chen et al. Nat. Commun. 11, 1202 (2020)] and even up to $\sim 2.5\,\mathrm{GV/m}$ [Taychatanapat et al. PRL 105, 166601 (2010)], which significantly exceed the fields we found in our experiment ($\sim 1.3\,\mathrm{GV/m}$). Thus, our observed effects are well-accessible in device geometries, highlighting the importance of our work for potential applications. We have added this discussion in the Conclusion section of the manuscript:

"The required interfacial fields are readily achievable in state-of-the-art electronic devices [49–51], highlighting the technological potential of our findings."

5) Magnetic and electric field tunable orbital magnetic moments have been seen before see works by Y. Lee et al. Physical Review Letters 2020, Z. Ge et al. Physical Review Letters 2021, and Z. Ge et al., Nature Nanotechnology 2023. The authors must include these prior works in their discussions and compare their findings with these prior works.

Reply: We agree with the reviewer that magnetic and electric field tunable orbital magnetic moments have been realized before in graphene and have included these important works in the paragraph mentioned above, under point 3). However, we would like to emphasize here that our observations do not rely on the lateral confinement of graphene states, i.e. quasi-0D systems. In contrast, the graphene/FGT interphase extends in 2D, making it relevant for devices that rely on the lateral transport of spin and orbital angular momentum between contacts. Together with the fact that the required electric fields are accessible in such devices, as discussed above, we believe that our work provides an important contribution to the field and warrants publication in Nature Communications.
