# Supplementary material for: Giant orbital Zeeman effects in a magnetic topological van der Waals interphase
Source: Nat Commun. 2026 Oct 23;17:11076. doi: 10.1038/s41467-026-78016-8 (PMC13694696; doi:10.1038/s41467-026-78016-8)
Supplement: Supplementary file 2 [file 41467_2026_78016_MOESM2_ESM.pdf]

Supplementary Information: Giant orbital Zeeman effects in a magnetic topological van der Waals interphase

Tobias Wichmann,[1,2,3] Mirco Sastges,[4,5,6] Keda Jin,[1,2,7] Jose Martinez-Castro,[1,2,7] Tom G. Saunderson,[4,5] Dongwook Go,[5,8] Honey Boban,[9] Samir Lounis,[10] Lukasz Plucinski,[9] Markus Ternes,[1,2,7] Yuriy Mokrousov,[4,5] F. Stefan Tautz[1,2,3] and Felix Lüpke[1,2,11,*]

[1]*Peter Grünberg Institute (PGI-3), Forschungszentrum Jülich, 52425 Jülich, Germany*
[2]*Jülich Aachen Research Alliance, Fundamentals of Future Information Technology, 52425 Jülich, Germany*
[3]*Institut für Experimentalphysik IV A, RWTH Aachen University, 52074 Aachen, Germany*
[4]*Institute of Physics, Johannes Gutenberg University, 55099 Mainz, Germany*
[5]*Peter Grünberg Institute (PGI-1), Forschungszentrum Jülich, 52425 Jülich, Germany*
[6]*Department of Physics, RWTH Aachen University, 52056 Aachen, Germany*
[7]*Institut für Experimentalphysik II B, RWTH Aachen University, 52074 Aachen, Germany*
[8]*Department of Physics, Korea University, Seoul 02841, Republic of Korea*
[9]*Peter Grünberg Institute (PGI-6), Forschungszentrum Jülich, 52425 Jülich, Germany*
[10]*Institute of Physics and Halle-Berlin-Regensburg Cluster of Excellence CCE, Martin-Luther-University Halle-Wittenberg, 06099 Halle, Germany*
[11]*Institute of Physics II, Universität zu Köln, Zülpicher Straße 77, 50937 Cologne, Germany*

*E-mail: f.luepke@fz-juelich.de

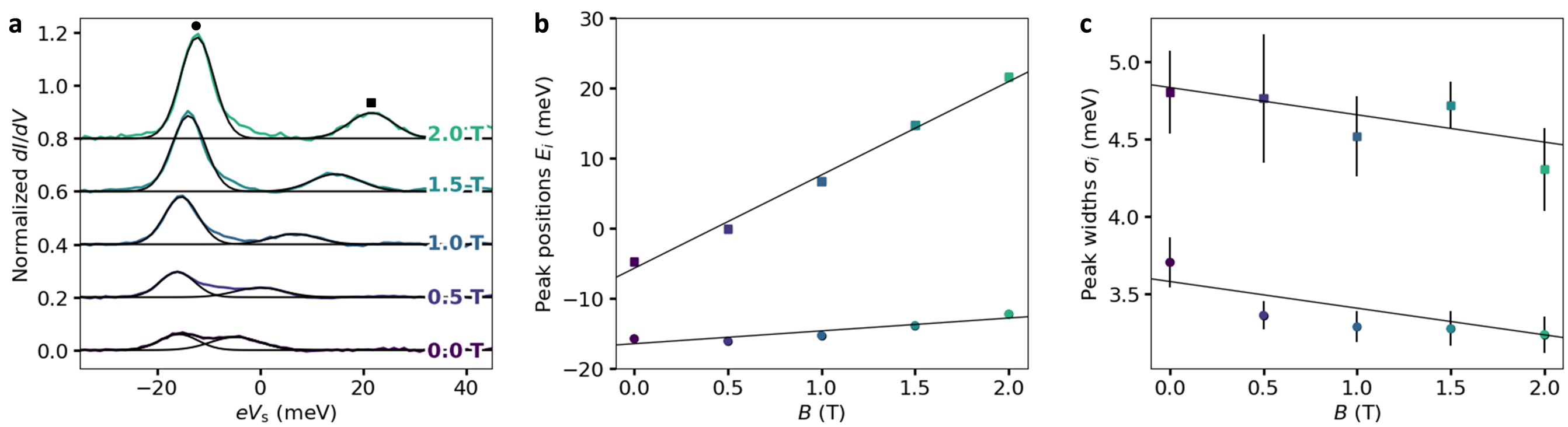

Supplementary Fig. 1. **Fit results for the two Fe_3GeTe_2-related peaks in the inelastic tunnelling gap of graphene, as a function of external *B* field.** **(a)** Differential conductance spectra (arbitrary units) after subtraction of a polynomial background (colored data curves, same data set as in Fig. 3b). The displayed Gaussians $A_i e^{-(eV_\mathrm{s}-E_i)^2/2\sigma_i^2}$ (black curves) were fitted to the data separately for each curve and peak, except at $B = 0$, where because of their significant overlap the two peaks were fitted simultaneously. **(b, c)** Fitted peak energies E_i and widths σ_i, respectively, with linear fits for each peak. Error bars correspond to the fit uncertainties.

I_t (nA)	δh (Å)	C_tip(µFcm^{-2})	E_D (meV)	n_gr (10^{10} cm^{-2})	E_FGT (GV/m)
0.1	0	0.885	-38.7 ± 2.6	5.50 ± 0.74	1.291 ± 0.008
0.3	0.500	0.932	-33.6 ± 2.6	4.15 ± 0.64	1.306 ± 0.008
0.5	0.732	0.955	-30.5 ± 2.2	3.42 ± 0.49	1.316 ± 0.007
0.8	0.946	0.978	-26.5 ± 2.7	2.58 ± 0.53	1.328 ± 0.008
1.0	1.048	0.989	-27.8 ± 2.4	2.84 ± 0.49	1.324 ± 0.007

Supplementary Table I. Change in tip height δh, tip-graphene capacitance C_tip (Eq. 13), Dirac point energy E_D (Fig. 4a), charge carrier concentration (Eq. 24), and interface electric field E_FGT (Eq. 1), all as a function of setpoint tunnel current I_t.

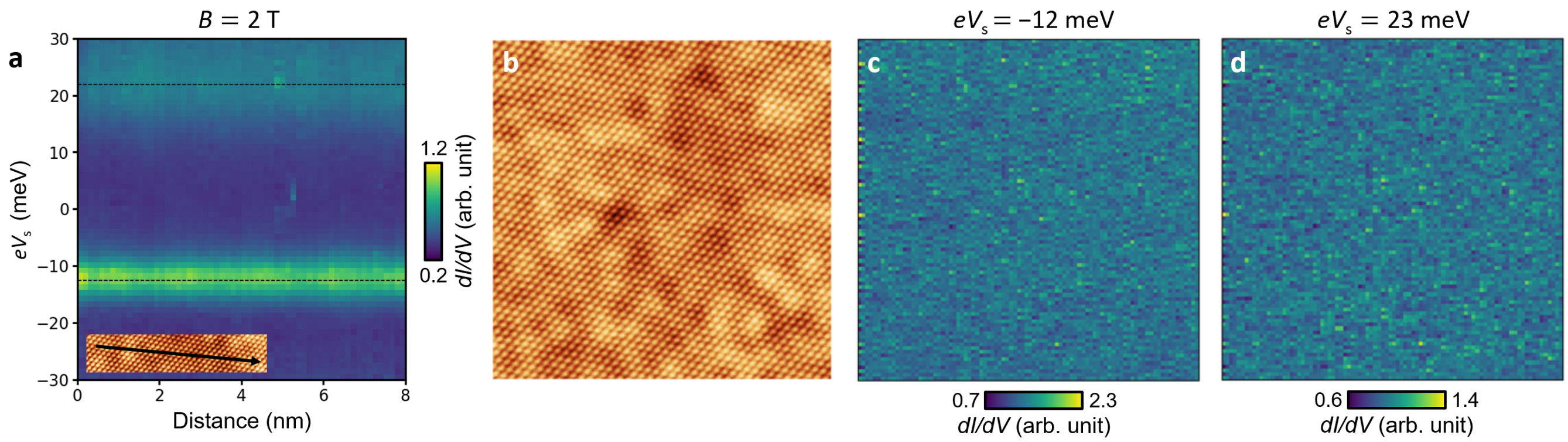

Supplementary Fig. 2. **Spatial consistency of the two Fe_3GeTe_2-related peaks at *B* = 2 T.** **(a)** Color map of differential conductance spectra (arbitrary units), recorded at positions along the arrow in the STM topography in the inset The map reveals constant peak energies and intensities. **(b)** STM topography recorded at another position on the graphene/Fe_3GeTe_2 heterostructure. Scan size: $10\,\mathrm{nm}$. **(c, d)** Color maps of differential conductance spectra (arbitrary units) recorded in the area shown in panel b at the two peak energies marked by dotted lines in panel a. No spatial variations, for example due to scattering at defects, are discernible. Tip stabilization parameters for all panels: $V_\mathrm{s} = 50\,\mathrm{mV}$, $I_\mathrm{t} = 1\,\mathrm{nA}$.

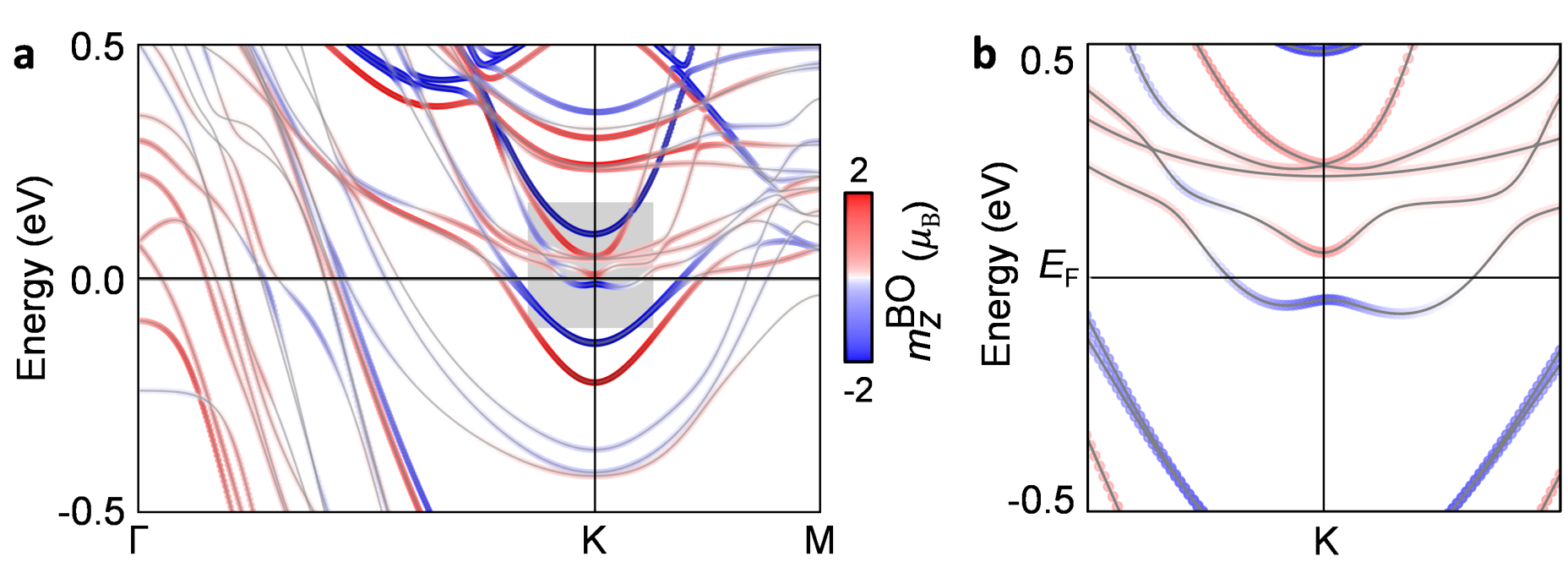

Supplementary Fig. 3. **DFT-calculated band structure of trilayer Fe_3GeTe_2.** **(a)** In Fe_3GeTe_2 trilayers, the inversion symmetry of bulk Fe_3GeTe_2 (bilayer unit cell) is broken. This leads to additional bands close to E_F, a smaller nodal line gap, and smaller band orbital moments (color coded). Furthermore, the K and K' points are no longer equivalent, although their differences are minimal. We thus only show the K point here. The band structure in this panel should be compared with Fig. 1c, which was calculated for bulk Fe_3GeTe_2. We note that the band crossing at K/K' does not exist in monolayer FGT. However, the spin canting, which is strongest in the layer closest to the interface (compare Fig. 1e) may break inversion symmetry for two or more FGT layers and thus can explain a smaller gap at K/K'. **(b)** Zoom into the shaded region in panel a, showing the bands close to the K point.

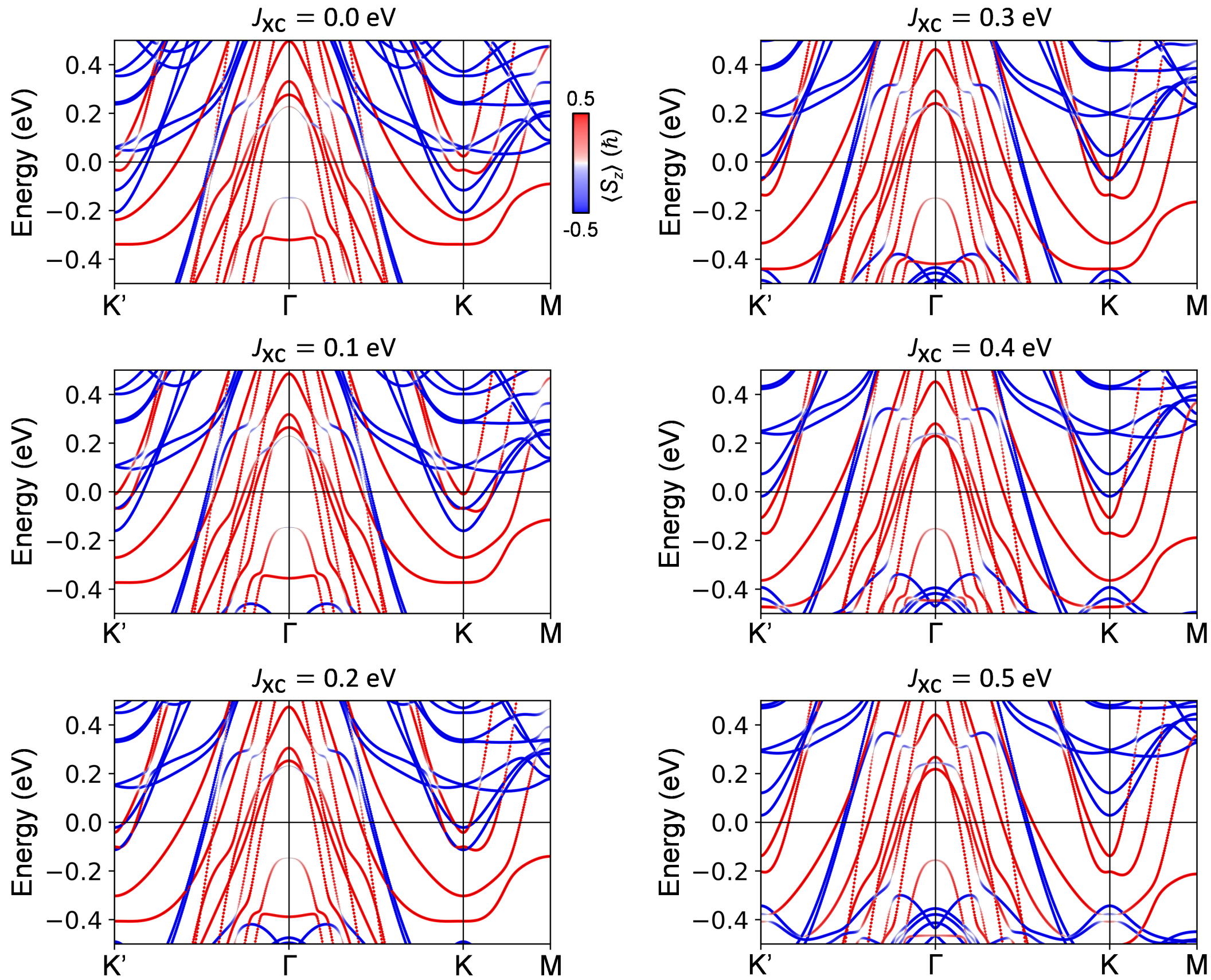

Supplementary Fig. 4. **Fe_3GeTe_2 band structure as a function of magnetic exchange potential strength.** To model the spin Zeeman effect in an external magnetic field, we took the DFT-calculated single-particle band structure of Fe_3GeTe_2 (see Fig. 1c) and added exchange coupling of varying size, ranging from $J_{\mathrm{xc}} = 0$ to $0.5\,\mathrm{eV}$. The additional exchange coupling is added on top of the FGT Wannier Hamiltonian by using the matrix elements of the spin operator in terms of Wannier functions.

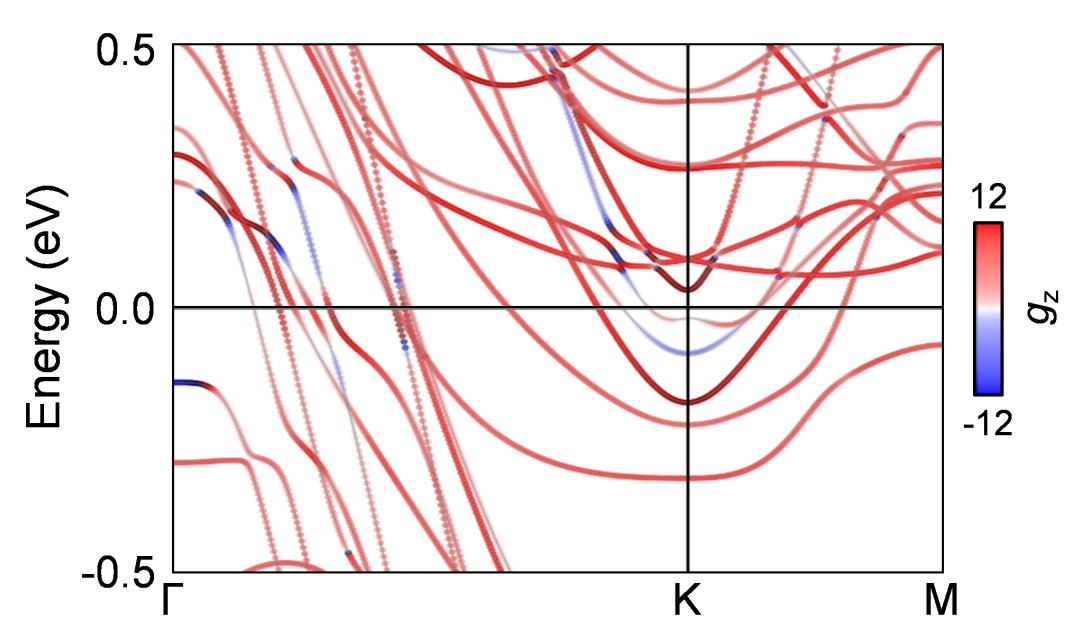

Supplementary Fig. 5. **Calculated out-of-plane *g*-factor, neglecting the influence of spin canting and chiral orbital moments.** The plot shows the band structure of Fig. 1c, with color-coded $g_z^{\mathrm{BO}} = 2 + \langle L \rangle / \langle S \rangle$ resulting from the spin and orbital moments shown as color-coding in Fig. 1c and d, respectively.

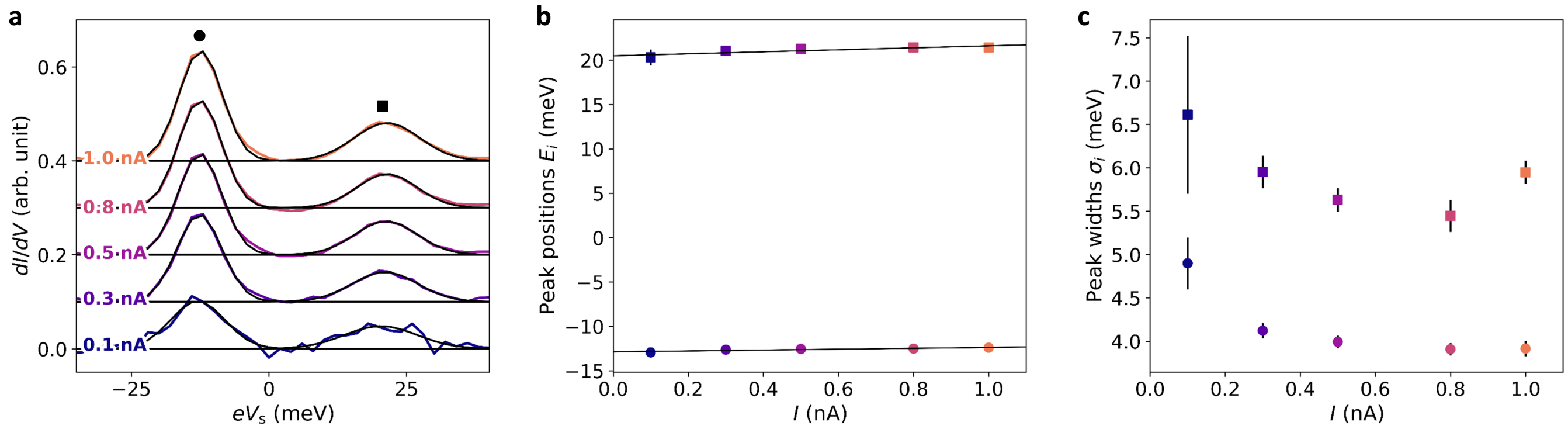

Supplementary Fig. 6. **Fit results for the two Fe_3GeTe_2-related peaks in the inelastic tunnelling gap of graphene, as function of setpoint current.** **(a)** Differential conductance spectra (arbitrary units, measured at $B = 2\,\mathrm{T}$) after subtraction of a polynomial background (colored data curves, same data set as in Fig. 4c). The displayed Gaussians $A_i e^{-(eV_\mathrm{s}-E_i)^2/2\sigma_i^2}$ (black curves) were fitted to the data separately for each curve and peak. **(b, c)** Fitted peak energies E_i and widths σ_i, respectively, with linear fits in panel b for each of the two peaks. Error bars correspond to the fit uncertainties.

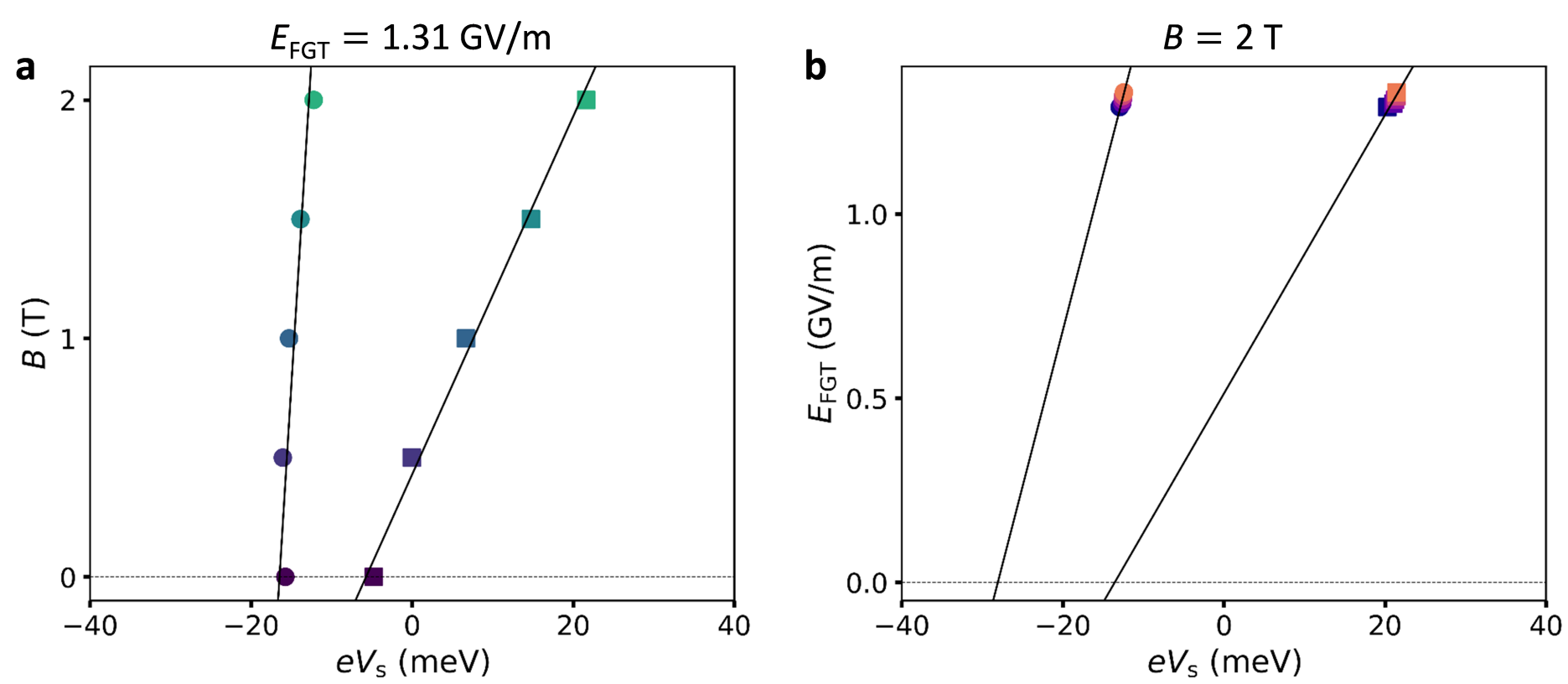

Supplementary Fig. 7. **Fitted peak positions for the two Fe_3GeTe_2-related peaks in the inelastic tunnelling gap of graphene, as function of magnetic and electric field.** **(a)** B-field-dependent Zeeman effect at $E_\mathrm{FGT} = 1.31\,\mathrm{GV/m}$. Data points and linear fits (solid lines) are the same as in Fig. 3c. **(b)** E_FGT-field-dependent Zeeman effect at $B = 2\,\mathrm{T}$. Data points and linear fits (solid lines) are the same as in Fig. 4d.

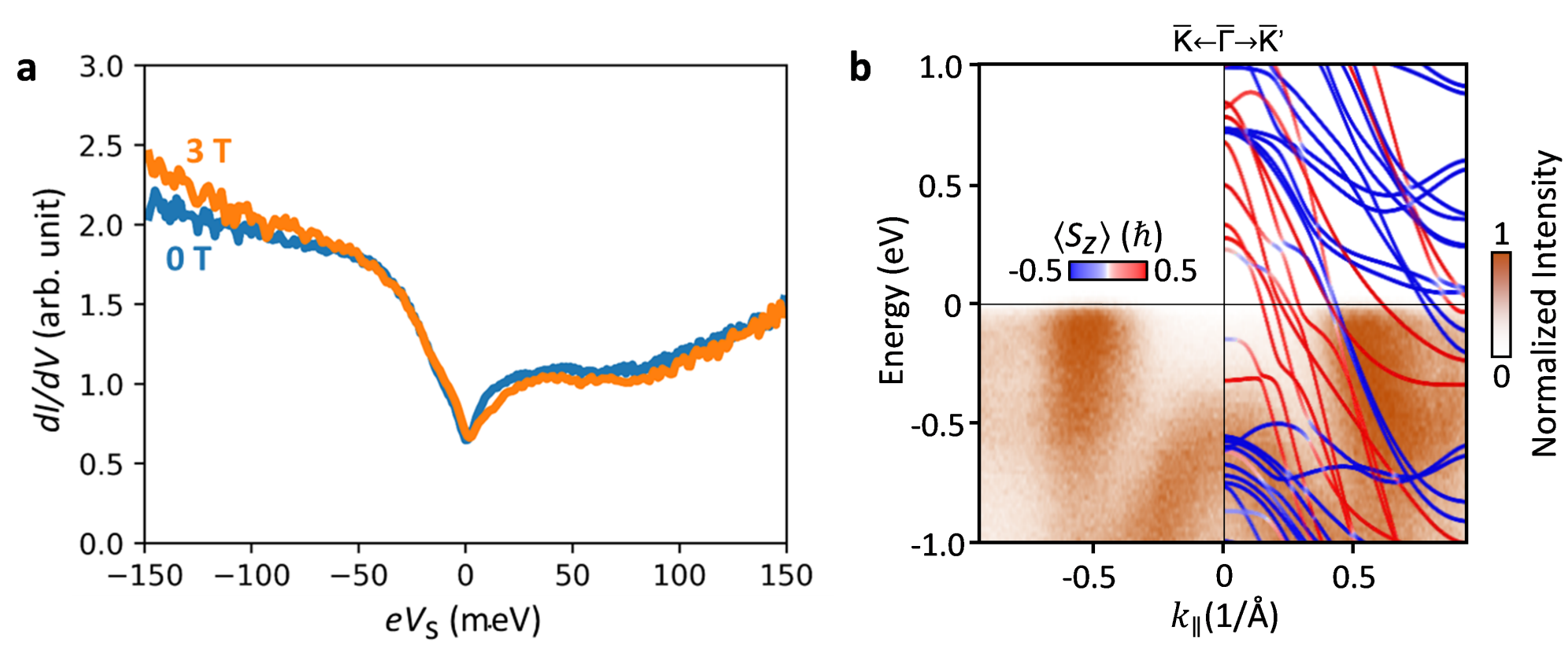

Supplementary Fig. 8. **Characterization of bulk Fe_3GeTe_2.** **(a)** Scanning tunneling spectra recorded on a bulk Fe_3GeTe_2 crystal. No significant change is observed as a function of applied magnetic field up to our maximum field of $B = 3\,\mathrm{T}$. The data is consistent with previous reports in the literature [1, 2] and confirms that breaking of inversion symmetry at the interface is a necessary condition to observe the specific behavior of spectral features reported in the main text. **(b)** Angle-resolved photoemission spectroscopy of a bulk Fe_3GeTe_2 crystal (band maps from $\bar{\Gamma}$ to $\bar{\mathrm{K}}$ on the left and $\bar{\Gamma}$ to $\bar{\mathrm{K}}'$ on the right) are overlayed with the DFT-calculated band structure including SOC (from Fig. 1c) in $\bar{\Gamma}-\mathrm{K}$ direction. Red and blue bands correspond to the majority (minority) spins.

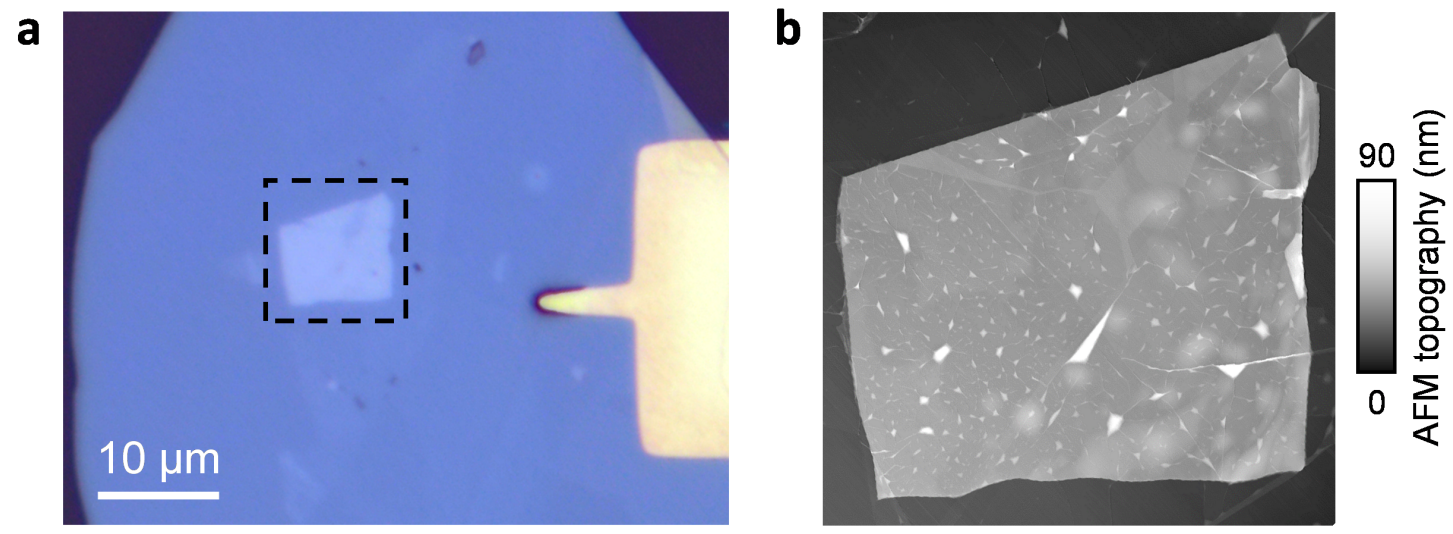

Supplementary Fig. 9. **Atomic force microscopy on the the graphene/Fe_3GeTe_2 heterostructure.** **(a)** Optical micrograph of the heterostructure (same as in Fig. 1e), with the black dashed line representing the outline of the AFM measurement in panel b. **(b)** Atomic force micrograph of the graphene-covered Fe_3GeTe_2 flake. The formation of 'dirt pockets' indicates an atomically clean vdW interface in between the pockets, in consistency with the spatially uniform STM measurements that were performed in different spots on the sample.

[1] M. Zhao, B.-B. Chen, Y. Xi, Y. Zhao, H. Xu, H. Zhang, N. Cheng, H. Feng, J. Zhuang, F. Pan, *et al.*, Kondo holes in the two-dimensional itinerant ising ferromagnet Fe_3GeTe_2, Nano Letters **21**, 6117 (2021).
[2] M. Zhao, Y. Zhao, Y. Xi, H. Xu, H. Feng, X. Xu, W. Hao, S. Zhou, J. Zhao, S. X. Dou, *et al.*, Electric-field-driven negative differential conductance in 2D van der Waals ferromagnet Fe_3GeTe_2, Nano Letters **21**, 9233 (2021).
